# 250-year records of mercury and trace element deposition in two lakes from Cajas National Park, SW Ecuadorian Andes

**DOI:** 10.1007/s11356-020-11437-0

**Published:** 2020-12-05

**Authors:** Tobias Schneider, Benjamin A. Musa Bandowe, Moritz Bigalke, Adrien Mestrot, Henrietta Hampel, Pablo V. Mosquera, Lea Fränkl, Giulia Wienhues, Hendrik Vogel, Wojciech Tylmann, Martin Grosjean

**Affiliations:** 1 Oeschger Centre for Climate Change Research, University of Bern, Hochschulstrasse 4, 3012 Bern, Switzerland; 2 Institute of Geography, University of Bern, Hallerstrasse 12, 3012 Bern, Switzerland; 3 Department of Geosciences, University of Massachusetts Amherst, 611 North Pleasant Street, Amherst, MA 01003-9297 USA; 4 Multiphase Chemistry Department, Max Planck Institute for Chemistry, Hahn-Meitner-Weg 1, 55128 Mainz, Germany; 5 Facultad de Ciencias Químicas, Universidad de Cuenca, Cuenca, Ecuador; 6 Laboratorio de Ecología Acuática, Departamento de Recursos Hídricos y Ciencias Ambientales, Universidad de Cuenca, Cuenca, Ecuador; 7 Subgerencia de Gestión Ambiental, Empresa Pública Municipal de Telecomunicaciones, Agua potable, Alcantarillado y Saneamiento (ETAPA EP), Cuenca, Ecuador; 8 Departament de Biologia Evolutiva, Ecologia i Ciències Ambientals, Universitat de Barcelona, Barcelona, Spain; 9 Institute of Geological Sciences, University of Bern, Baltzerstrasse 1+3, 3012 Bern, Switzerland; 10 Faculty of Oceanography and Geography, University of Gdansk, Bazynskiego 4, 80309 Gdansk, Poland

**Keywords:** Mercury, Trace elements, Heavy metals, Environmental reconstruction, Lake sediments, Paleolimnology, Anthropocene, Andes

## Abstract

Historical records of trace elements in lake sediments provide source-to-sink information about potentially toxic pollutants across space and time. We investigated two lakes located at different elevations in the Ecuadorian Andes to understand how trace element fluxes are related to (i) geology, (ii) erosion in the watersheds, and (iii) local point sources and atmospheric loads. In remote Lake Fondococha (4150 m a.s.l.), total Hg fluxes stay constant between ca. 1760 and 1950 and show an approximately 4.4-fold increase between pre-1950 and post-1950 values. The post-1950 increase in fluxes of other trace elements (V, Cr, Co, Ni, Cu, Zn, As, Cd, and Pb) is lower (2.1–3.0-fold) than for Hg. Mostly lithogenic sources and enhanced soil erosion contribute to their post-1950 increase (lithogenic contribution: > 85%, Hg: ~ 58%). Average post-1950 Hg fluxes are approximately 4.3 times higher in peri-urban Lake Llaviucu (3150 m a.s.l.) than in the remote Lake Fondococha. Post-1950 fluxes of the other trace elements showed larger differences between Lakes Fondococha and Llaviucu (5.2 < 25–29.5-fold increase; Ni < Pb–Cd). The comparison of the post-1950 average trace element fluxes that are derived from point and airborne sources revealed 5–687 (Hg–Pb) times higher values in Lake Llaviucu than in Lake Fondococha suggesting that Lake Llaviucu’s proximity to the city of Cuenca strongly influences its deposition record (industrial emissions, traffic, caged fishery). Both lakes responded with temporary drops in trace element accumulations to park regulations in the 1970s and 1990s, but show again increasing trends in recent times, most likely caused by increase in vehicular traffic and openings of copper and gold mines around Cajas National Park.

**Supplementary Information:**

The online version contains supplementary material available at 10.1007/s11356-020-11437-0.

## Introduction

Trace elements (here V, Cr, Co, Ni, Cu, Zn, As, Cd, Pb, and Hg) in the environment are a global concern, due to their potential toxicity to living organisms (Tchounwou et al. 2012; Beal et al. 2014; Sundseth et al. 2017) and their effects on environmental processes (Chojnacka 2018). They follow different pathways in the atmosphere, the hydrosphere and on land, depending on their physicochemical characteristics (Rauch and Pacyna 2009; Beal et al. 2013; Driscoll et al. 2013). Most trace elements in the environment originate from natural sources (e.g., volcanic activity) and from anthropogenic emissions (Konieczka et al. 2018). Anthropogenically derived trace elements have specific spatially and temporally explicit trajectories of production rates from prehistoric to industrial times (e.g., mining and amalgamation, fossil fuel combustion, and industrial emissions; Streets et al. 2011; Cooke and Bindler 2015). The least volatile elements can be bound to large particles and are mostly deposited close to their point sources whereas the more volatile ones (gaseous; such as Hg) may be transported over long distances in the atmosphere (Schroeder and Munthe 1998; Phillips et al. 2011). This leads to pronounced spatial and temporal variability of depositional rates across the world (Selin et al. 2008; Strode et al. 2009; Streets et al. 2011; Engstrom et al. 2014; Cooke and Bindler 2015).

Lake sediments are very valuable environmental archives for documenting past deposition of trace elements and Hg from natural and anthropogenic activities (Smol 2008; Boyle et al. 2015). Most of the high-resolution Hg records span the ^210^Pb dating range of the past 150 years, and the study sites are predominantly located in the mid- and high-latitude areas of the northern hemisphere, with very few records in the southern hemisphere mid-latitudes and the inner tropics (Perry et al. 2005; Biester et al. 2007; Cooke et al. 2009; Beal et al. 2013; Beal et al. 2014; Engstrom et al. 2014; Álvarez et al. 2018; Engels et al. 2018; Guédron et al. 2018; Guédron et al. 2019). A majority of the sediment records from the northern hemisphere reveal an overall 2–5-fold increase of Hg deposition rates from pre-industrial to modern (1950–2000+) times (Fitzgerald et al. 1998; Landers et al. 1998; Perry et al. 2005; Biester et al. 2007; Mast et al. 2010; Pirrone et al. 2010; Beal et al. 2013; Engstrom et al. 2014) and provide critically important information about “natural” background and anthropogenic depositional rates for times long before direct measurements became available. Moreover, reconstructions provide data to constrain global Hg models (Strode et al. 2009). All this, in combination, makes it important to understand depositional rates (fluxes) across the world and through time, particularly in remote high-mountain and arctic environments (Schroeder and Munthe 1998; Phillips et al. 2011).

Refining the network of historical trace element and Hg flux records across the globe remains a coveted goal. Specific goals include among other (i) assessing pre-anthropogenic (natural) background levels of potentially toxic trace elements, (ii) assessing the differences between the historical legacy (here restricted to the period 1760–1950) and the modern era (post-1950) in terms of the levels and composition profiles of trace elements, and (iii) the apportionment of sources, i.e., differentiation of local sources (e.g., from the catchment, and point sources) from sources related to long-range atmospheric transport (Perry et al. 2005).

Here, we present the depositional history of Hg and other trace elements (V, Cr, Co, Ni, Cu, Zn, As, Cd, and Pb) as recorded in lake sediments from Cajas National Park (CNP) in the high Andes of southwestern Ecuador. CNP forms part of the UNESCO Biosphere Reserve. It is recognized for its biodiversity and wetlands and is considered as one of the most pristine areas in the high Andean grasslands, the Páramo. The high Andean CNP provides approximately 60% of the citizens of the city of Cuenca with fresh water (Mosquera et al. 2017). However, the Ecuadorian Ministry of Mining granted exploratory mining concessions and, thus, large areas around the park have recently become active registered mining sites and large-scale gold mines are currently under construction (Roy et al. 2018).

In the present study, we compare two nearby lakes: Lake Llaviucu (3150 m a.s.l.), a peri-urban lake in the vicinity of the city of Cuenca and surrounded by montane forest, and remote Lake Fondococha (4130 m a.s.l), a high-elevation lake in the Páramo grassland. In these same lakes, Bandowe et al. (2018) have established that fluxes of polycyclic aromatic compounds (PACs) are 4–5 times higher in the lower-elevation peri-urban Lake Llaviucu than in remote high-elevation Lake Fondococha. They concluded that the cloud condensation maximum (3500 m a.s.l.) in combination with denser vegetation seem to scavenge and remove organic pollutants at lower elevations very efficiently from the atmosphere. Furthermore, they found that remote Lake Fondococha (above the cloud-layer maximum) shows a PAC profile with much larger contributions of long-distance transported pollutants, whereas the sources for PACs in the lower peri-urban Lake Llaviucu are mostly locally derived (traffic, combustion, and industrial emissions from Cuenca; Bandowe et al. 2018). Yet, no study from this region has investigated whether similar mechanisms apply for the deposition of Hg and other trace elements.

Guided by the scientific challenges mentioned above, we addressed the following questions in the present study: (i) What is the temporal pattern of Hg and other trace elements deposition in two lakes (high-altitude and remote, and lower-altitude and located in peri-urban area) over the past approximately 250 years? Are the source patterns for Hg (mostly gaseous) different from other trace elements that are mostly bound to particulate aerosols (following the apportionment approach from Perry et al. 2005)? (ii) What is the role of anthropogenic emissions and increased erosion in the catchment (comparing post-1950 to pre-1950 conditions), and which part may be attributed to point sources (directly in the catchment, and in the lake), local sources from the city of Cuenca, or long-distance atmospheric transport?

## Material and methods

### Regional setting

**Fig. 1:**
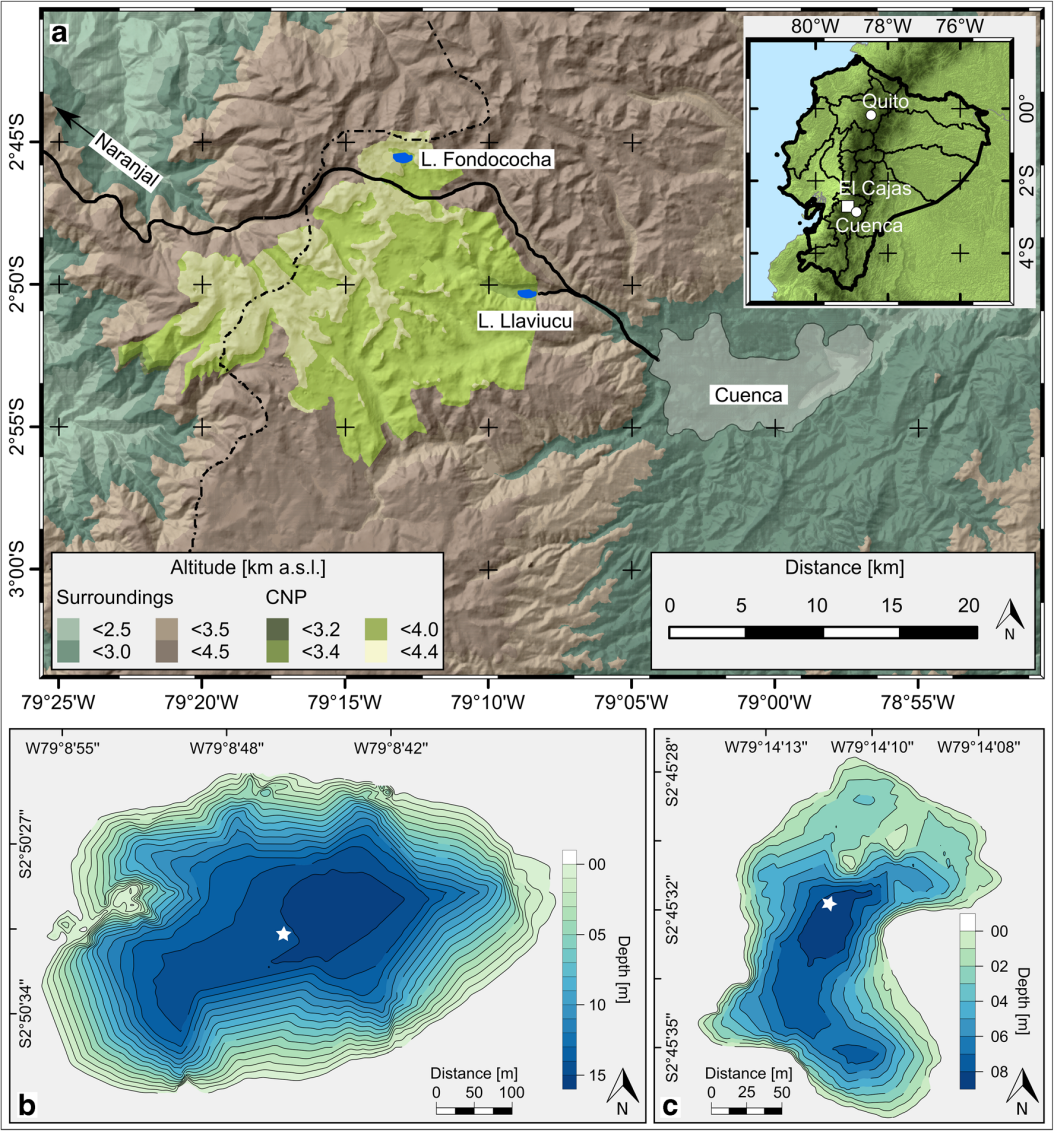
Geographic location of Lakes Llaviucu and Fondococha in Cajas National Park. **a** Cajas National Park (green shaded area) in southern Ecuador with the continental divide (dashed line), the highway (black line), and the city of Cuenca. Bathymetric maps for Lake Llaviucu (**b**) and Lake Fondococha (**c**) with the coring sites (white asterisks). (Adapted from Bandowe et al. 2018)

Both studied lakes are located in the Cajas National Park (CNP; 2° 50′ S, 79° 10′ W, Fig. 1) on the eastern slope of the western Andean Cordillera in the Azuay Province, southern Ecuador. Approximately 80% of the park area belongs to the Pleistocene Tarqui formation (tuff, rhyolite, and andesite; Paladines et al. 1980). CNP is also exposed to the ashfall trajectories of different still active volcanoes (Arcusa et al. 2020). The soils in the watersheds are therefore mostly of volcanic origin (non-allophanic Andosols and Histosols) and are characterized by a high organic matter content (Mosquera et al. 2017).

Lake Fondococha (2° 45′ 35″ S, 79° 14′ 11″ W) is a small (3.4 ha) oligotrophic remote postglacial lake at high-elevation (4130 m a.s.l.) in the Páramo grasslands, substantially above the precipitation maximum which is at 3500 m a.s.l (Schneider et al. 2018). The lake is 9.9 m deep and the catchment is relatively small (0.6 km^2^, catchment area/lake area ratio = 18; Mosquera et al. 2017).

Lake Llaviucu (2° 50′ 36″ S, 79° 8′ 46″ W), in contrast, is a mesotrophic exorheic lake, dammed by a terminal moraine (ca. 35,000 cal. years BP; Colinvaux et al. 1997) with a surface area of 18.9 ha and a maximum depth of 16.5 m. The lake is located at relatively lower elevation (3140 m a.s.l.) in the montane rain forest belt below the precipitation maximum and the cloud condensation level (3500 m a.s.l). The distance to Lake Fondococha is approximately 13.5 km (NW) and Lake Llaviucu is close to the suburbs of Cuenca (< 10 km). The Cuenca-Molleturo-Naranjal highway (Fig. 1) passes within 2 km of the lake and an intensive caged fishery was established in the lake between 1978 and 1998 (data provided by ETAPA EP). The catchment of Lake Llaviucu (~47.7 km^2^) is substantially larger and ranges up to the Páramo belt (catchment area/lake area ratio = 252; Mosquera et al. 2017).

The area of CNP, including the catchments of both lakes, has been subject to human activities since the late Holocene (since ca. 4000 cal. years BP; Hansen et al. 2003). Deforestation, grazing, and grassland burning was particularly intense in the second half of the twentieth century before the founding of the recreational area (1977) and the National Park (1996). Bandowe et al. (2018) concluded from ^210^Pb dated sediment cores of both lakes that sediment mass accumulation rates (MARs) were remarkably constant prior to 1960 but rapidly increased afterwards by a factor of 2, reflecting mostly soil erosion from the catchment. High MARs (soil erosion) were observed until the late 1980s when they started to decrease slowly as human activities decreased with increasing environmental protection. Given the economic development of the city of Cuenca, we define here the “modern era” as post-1950 (phase of Great Acceleration; Steffen et al. 2015). Arcusa et al. (2020) recently identified several distinguished volcanic ash layers (macro tephra layers) in the sediments of both studied lakes. However, no tephra layers were detected in the last approximately 250 years.

### Sediment sampling and chronostratigraphy

In 2014, annually integrating sediment traps (July 2014–July 2015) were deployed at the depocenter and short sediment cores were retrieved from the depocenter of the two lakes (Fig. 1) using a gravity coring system (UWITEC, Austria). The sediment-water interfaces of the retrieved sediment cores were preserved with wet floral foam to guarantee safe shipment. The cores were kept at dark and cool (4 °C) conditions until they were split lengthwise in the laboratory, sedimentologically described, and photographed (Schnurrenberger et al. 2003). Prior to physical subsampling, the half cores were scanned with an μXRF-system (ITRAX; Croudace et al. 2006) equipped with a Mo-tube. The exposure time was set to 10 s, the current to 35 mA, and the voltage to 30 kV. One core half per core (FON14-1 and LLA14-1) was stratigraphically sampled at 0.5-cm increments down to 12 cm in Lake Fondococha (lower sedimentation rate; Bandowe et al. 2018) and down to 35 cm in Lake Llaviucu (higher sedimentation rate; Bandowe et al. 2018) for Hg and trace element analyses. The concentrations of carbon, nitrogen, and sulfur (C, N, S), and polycyclic aromatic compound (PACs) data in these same sediments were adopted from a previous study (Bandowe et al. 2018). Radionuclide samples (^210^Pb and ^226^Ra) used for the chronologies were determined at the same intervals on the parallel core halves in a previous study (Bandowe et al. 2018), following the methods described in Tylmann et al. (2016). After testing varying age-depth model assumptions (von Gunten et al. 2009), we decided to use the constant rate of supply (CRS) age-depth models (Appleby and Oldfield 1978) with missing inventory corrections (Tylmann 2014) to calculate sediment ages and mass accumulation rates (MAR). Here, we adopt the sediment ages and MARs as published in Bandowe et al. (2018) and projected them further back in time (Lake Fondococha: ca. 1760; Lake Llaviucu: ca. 1780) based on linear extrapolation of the last four measured samples (Lake Fondococha: 1857–1890; Lake Llaviucu: 1891–1908).

All statistical analyses were conducted in R (R Core Team 2019) using the “vegan” (Oksanen et al. 2019), “factoextra” (Kassambara and Mundt 2017), and “corrplot” (Wei and Simko 2017) packages.

### Mercury and trace element analyses

One hundred milligrams of lyophilized and homogenized sediment (per sediment core sample and sediment trap sample) were digested in Teflon vessels with 8 ml HNO_3_ (69%, grade “supra pure”) and 2 ml H_2_O_2_ (30%, grade “supra pure”) in a microwave oven (Ethos contFLOW1600, Milestone, Shelton CT, USA, detailed program in Tab. S1). The digests were split into two portions, one was stabilized with HCl and used for Hg analysis and the other one was analyzed for the other trace elements. Each microwave batch included eight sediment samples in separate vessels, one vessel containing certified reference material (CRM, 2709a San Joaquin Soil, National Institute of Standards and Technology; Mackey et al. 2010), and one blank vessel (only with 8 ml HNO_3_ and 2 ml H_2_O_2_).

The trace element and Hg concentrations in digests were determined using an inductively coupled plasma mass spectrometer (ICP-MS; 7700x, Agilent Technologies, Santa Clara, USA). ICP-MS is an analytical technique commonly used to analyze Hg and other trace elements (D’Ilio et al. 2010; Djedjibegovic et al. 2012). Mercury and the other trace elements were measured separately with optimized operating parameters (Tab. S2). Rhodium and indium were used as instrumental internal standards. To avoid carry-over effects (“memory-effects”; Li et al. 2006), the Hg samples and standards were diluted to concentrations lower than 2 μg l^−1^ and, furthermore, after running each sample the system was rinsed with an (i) alkaline solution (EDTA Triton X), an (ii) acid solution (5% HNO_3_, 5% HCl), and also (iii) the sample’s matrix solution (1% HNO_3_, 0.5% HCl). The maximum blank values varied between 1.126 μg L^−1^ for Zn and 0.004 μg L^−1^ for Hg (Tab. S3).

The limit of detection (LOD) was calculated for every element (Tab. S3). We applied recovery corrections to the concentration measurements to estimate the effects of different microwave batches on the temporal trends of the concentrations (details presented in Tab. S5). The average recoveries per element ranged between 66 and 118% (U, Mn; Lake Fondococha) and 49 and 87% (Al, Fe; Lake Llaviucu) of the CRM 2709a values (we refer to their Table 3). A comparison of recovery corrected with not-recovery corrected concentration data revealed that their relative (temporal) trends are similar, and therefore, we decided to base further calculations on the not-recovery corrected concentration values. The reproducibility of the method was tested by replicate digests and analysis of selected samples (3 triplicates, 2 duplicates). The relative standard deviations (RSD) of the replicates ranged in average from 7% (Pb) to 16% (Cr; Tab. S4).

### Apportionment of elemental fluxes

In principle, lake sediments integrate matter fluxes (e.g., trace elements) from different sources. To further interpret the causes of trace element accumulation, it is necessary to disentangle the contribution of the different sources to the total fluxes per element. It is particularly important to correct for the impact of lithogenic inputs (e.g., soil erosion; Hermanns and Biester 2013) on the total flux per element. One procedure is to normalize the concentrations of elements of interest with the concentrations of inert trace elements (e.g., Al, Zr, Ti; Hermanns and Biester 2013). However, due to low recoveries of Al, Zr, and Ti (elements typically used for such purposes), we followed a different approach. Perry et al. (2005) proposed to apportion the total flux of Hg (*F*_tot_) to the lake sediments into (i) a fraction that reflects pre-anthropogenic “background” levels (*F*_B_); (ii) a fraction (*F*_V_) that is attributable to variations in soil erosion and lithogenic influx and, thus, mainly controlled by MAR; and (iii) a fraction (*F*_A_) which is attributable to direct atmospheric fallout and non-lithogenic point sources (Perry et al. 2005). *F*_B_ and *F*_V_ are lake-specific and depend mostly on bedrock geology, “natural” erosion, and erosion due to anthropogenic activities in the watersheds. Perry et al. (2005) pointed out that MAR and, in consequence, *F*_tot_, *F*_B_, *F*_V_, and *F*_A_ are highly dependent on the ^210^Pb-chronology. A correction for sediment focusing as applied in Perry et al. (2005) was not possible as there is no regional standard ^210^Pb profile (sedimentary inventory) available in the CNP. Here, we applied this apportionment procedure to the other trace elements too. We calculated elemental fluxes (*F*) at depth *i* as *F*_*i*_ = *C*_*i*_ × MAR_*i*_, where *C*_*i*_ is the concentration of the trace element and MAR the mass accumulation rate. Following Perry et al. (2005), we defined “background” trace element flux (*F*_B_) as the average pre-1900 flux (to exclude bias, we compared pre-1900 also to pre-1800 and pre-1850, all three produced similar values), and *F*_V*i*_ as the fluxes at sediment depth *i* attributable to variable MAR (assuming constant sediment composition; *F*_V*i*_ = {(MAR_*i*_ / MAR_pre-1900_ × *F*_B_) − *F*_B_}). *F*_tot_ is defined as the total flux at sediment depth *i*. *F*_A*i*_ is defined as the flux attributable to atmospheric or non-lithogenic point sources to the lake at sediment depth *i* (*F*_A*i*_ = *F*_tot_ − *F*_V*i*_ − *F*_B_).

## Results

### General characteristics of sediments

**Fig. 2:**
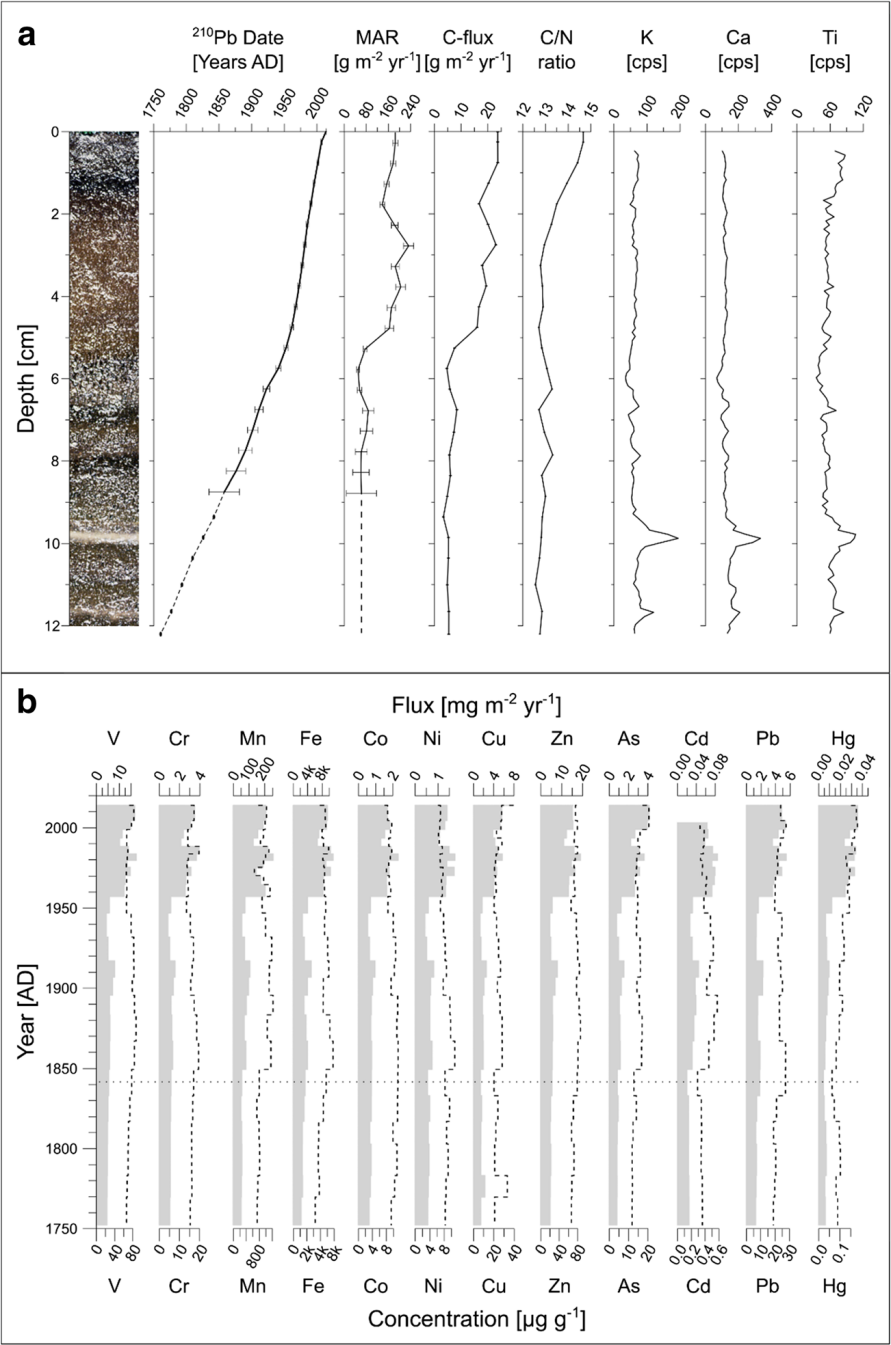
Chronology and trace element stratigraphy of Lake Fondococha. **a** Core image of Lake Fondococha with the ^210^Pb age-depth model (dashed line represents the extrapolated age-depth model, based on the lowest four samples), C fluxes, C/N ratios and selected μXRF-elements plotted along core-depth and **b** concentrations (dashed line) and fluxes (shaded) of discussed trace elements. The dotted horizontal line represents the start of the period based on the extrapolated age-depth model. Panel **a** was modified from Bandowe et al. (2018)

**Fig. 3:**
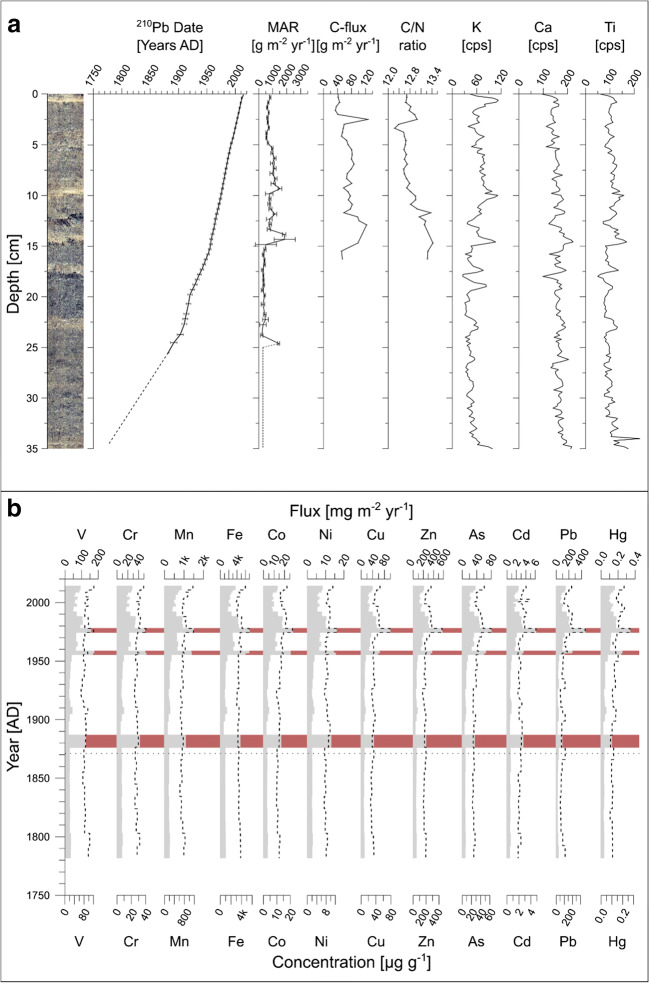
Chronology and trace element stratigraphy of Lake Llaviucu. **a** Core photograph of Lake Llaviucu with the ^210^Pb age-depth model (dashed line represents the extrapolated depth-age model, based on the lowest four samples), C fluxes, C/N ratios, and selected μXRF-elements plotted along core-depth and **b** concentrations (dashed line) and fluxes (shaded) of discussed trace elements. The dotted horizontal line represents the start of the period based on the extrapolated age-depth model. The red shadings in panel **b** highlight for the calculations excluded outliers (caused by high MAR due to rapid instantaneous event layers, flood layers). Panel **a** was modified from Bandowe et al. (2018)

The visible characteristics of the background sediments (excluding the instantaneously deposited layers) from both lakes are very homogeneous throughout the investigated sediment core sections. They consist of brownish-black (HUE 10YR 2/1) organic diatomaceous fine silt with slightly darker and lighter bands (Figs. 2a and 3a). The lighter bands represent sediment sections with enhanced influx of lithogenic components. Scanning μXRF analysis (K, Ca, Ti; Figs. 2a and 3a) underline the relatively stable sedimentary composition and indicate the lithogenic layers (more details provided in Bandowe et al. 2018).

The mass accumulation rate (MAR; Figs. 2a and 3a) reconstructed from Lake Fondococha’s sediment core is up to five times lower than the one measured in the sediments of Lake Llaviucu. However, the general trends (except within the layers of rapid sedimentation) compare well and both profiles do reveal a rapid MAR increase starting at ca. 1950, a significant MAR decrease between 1985 and ca. 1992 and thereafter again a slight MAR increase towards 2014. In both lakes, C/N ratios are above 12 indicating both terrestrial and lacustrine origin of the organic material (Meyers 1994). In contrast to Lake Fondococha, where the C/N ratio increases towards more recent times, the C/N values in Lake Llaviucu show an overall decreasing trend since 1950 (more details provided in Bandowe et al. 2018).

### Trace element concentrations and fluxes

#### Trace element trends in Lake Fondococha since 1760 ad

The trace element concentrations in high-elevation and remote Lake Fondococha show remarkably constant values with only minor fluctuations between ca. 1760 and 2014 (Fig. 2b) and no (V, Cr, Co, Ni, Zn, Cd) or only slightly positive trends (Cu, As, Pb) from ca. 1760 to 2014. The ratio of the average concentration in post-1950 sediments to the average concentration in pre-1950 sediments ranged from 0.8 to 1.1 (Table 1, Fig. 2b). Manganese and Fe concentrations are at least one order of magnitude higher compared to the other elements and show no increasing trend over the investigated sequence (Mn: 0.97; Fe: 1.01; post-1950/pre-1950). Mercury shows a 1.6-fold post-1950 increase in concentrations compared to pre-1950 average values.

**Table 1:** Lake Fondococha: TE (trace element), concentrations (μg g^−1^_ds_) and fluxes (mg m^−2^ year^−1^) (pre-1950 and post-1950, not recovery corrected values, for recovery corrected values refer to Tab. S8), Factor (average in post-1950 sediments relative to the average in pre-1950 sediments), Recent (2 top samples, mean year: ad 2007), comparison with threshold effect concentrations (LEL—low effect level, Persaud et al. 1993; ERL—effect range-low, Long and Morgan 1991) from sediment quality guidelines (SQG) in MacDonald et al. (2000), and comparison with threshold values in agricultural soils (TAS) after Toth et al. (2016) for trace elements in Lake Fondococha (bold: concentrations exceed LEL values of SQG; italics: concentrations exceed ERL values of SQG)

TE	This study Concentration (μg g^−1^_ds_)				SQGs-LEL (μg g^−1^_ds_)		TAS (μg g^−1^_ds_)	This study Flux (mg m^−2^ year^−1^)			
	Pre-1950*	1950–2014	Factor	Recent	LEL	ERL		Pre-1950*	1950–2014	Factor	Recent
Cr	16.7	15.1	0.9	16.53	26	80	100	1.15	2.83	2.45	3.32
Ni	8.82	6.97	0.79	6.65	16	30	200	0.61	1.3	2.14	1.34
Cu	**24.67**	**24.79**	1	**33.4**	16	70	100	1.71	4.64	2.72	6.71
Zn	76.29	74.05	0.97	75.97	120	120	200	5.29	13.87	2.62	15.27
As	**14.16**	**15.83**	1.12	**18.93**	6	33	5	0.98	2.95	3.01	3.8
Cd	0.42	0.37	0.87	0.29	0.6	5	1	0.03	0.07	2.3	0.06
Pb	22.73	22.68	1	23.9	31	35	60	1.58	4.23	2.69	4.8
Hg	0.094	*0.153*	1.63	*0.17*	0.2	0.15	0.5	0.006	0.028	4.39	0.03
C**	79.95	102.44	1.3	117.89	NA		NA	6.07	19.04	3.1	23.7

*Includes data points pre 1850; **multiplied by 1000

The trace element concentrations are mostly below low effect levels (LEL, Persaud et al. 1993), i.e., typical threshold values from sediment quality guidelines (Table 1, MacDonald et al. 2000), except for Cu, and As, where the pre- and post-1950, and the most recent sediment concentrations (Cu: 24.7–33.4 μg g^−1^_ds_, As: 14.2–18.93 μg g^−1^_ds_) are 1.5–3 times higher than threshold values (Table 1).

The vertical concentration trends of As and Hg are positively correlated (*p* < 0.05) with the concentrations of total carbon and nitrogen (C, N; Pearson correlation matrix and PCA, Fig. S1), while Co (with C, N), Ni (with C, N), and Sr (with C) are significantly negatively correlated. The correlations between all other trace element concentrations and C and N concentrations were not significant.

All trace element fluxes, including Hg, remained nearly constant during the whole pre-1950 period (except for a synchronous temporary peak at around 1910) but increased rapidly from 1950 to 2014. The post-1950 fluxes were higher than the pre-1950 levels (ca. 1760–1950) by factors of 2.1 (Ni) to 4.4 (Hg, Table 1). Between 1950 and 1970 an initial increase to higher modern values took place. Most trace elements showed maximum fluxes between 1970 and 1985 and increased again after a local minimum at around 1990. The fluxes of all investigated elements stayed at relatively high levels over the recent two decades.

#### Trace element trends in Lake Llaviucu since 1760 ad

In contrast to the small and remote high-altitude Lake Fondococha, trace element concentrations show larger variability in the lower-altitude peri-urban Lake Llaviucu (Fig. 3b). They are relatively stable prior to 1950, increase thereafter showing peak values in the mid-1970s, decrease to a relative local minimum at around 2000, and increase again towards recent times. The average concentrations in post-1950 sediments relative to the average concentrations in pre-1950 sediments (ratios) range from 1.0 (Fe) to 1.9 (Pb; Fig. 3b, Table 2). These factors are higher than those seen in Lake Fondococha (0.8–1.1; Table 1). The concentrations of Cr, Cu, Zn, As, Cd, and Pb are the highest in these sediments and their values exceed the threshold values as specified in sediment quality guideline’s LEL (Persaud et al. 1993) by factors of 1.2 (Cr) to 6 (As) and in the most recent sediments by factors of 1.3 (Cr) to 7 (As; Table 2).

**Table 2:** Lake Llaviucu: TE (trace element), concentrations (μg g^−1^_ds_) and fluxes (mg m^−2^ year^−1^) (pre-1950 and post-1950, not recovery corrected values, for recovery corrected values refer to Tab. S9), Factor (average in post-1950 sediments relative to the average in pre-1950 sediments), Recent (2 top samples, mean year: ad 2011), comparison with threshold effect concentrations (LEL—low effect level, Persaud et al. 1993; ERL: effect range-low, Long and Morgan 1991) from sediment quality guidelines (SQG) in MacDonald et al. (2000), and comparison with threshold values in agricultural soils (TAS) after Toth et al. (2016) for trace elements in Lake Llaviucu (bold: concentrations exceed LEL values of SQG; italics: concentrations exceed ERL values of SQG)

TE	This study Concentration (μg g^−1^_ds_)				SQGs-LEL (μg g^−1^_ds_)		TAS (μg g^−1^_ds_)	This study Flux (mg m^−2^ year^−1^)			
	Pre-1950*	1950–2014	Factor	Recent	LEL	ERL		Pre-1950*	1950–2014	Factor	Recent
Cr	**27.1**	**29.8**	1.1	**34.41**	26	80	100	8.68	22.57	2.6	26.92
Ni	8.1	8.93	1.1	10.02	16	30	200	2.59	6.75	2.61	7.83
Cu	**31.73**	**40.67**	1.28	**46.35**	16	70	100	10.17	31.09	3.06	36.23
Zn	***178.8***	***226.41***	1.27	***253.68***	120	120	200	57.25	174.01	3.04	198.35
As	**25.87**	***35.84***	1.39	***42.23***	6	33	5	8.29	27.67	3.34	32.96
Cd	**2.11**	**2.61**	1.24	**2.94**	0.6	5	1	0.67	1.98	2.93	2.3
Pb	***76.82***	***141.94***	1.85	***173.37***	31	35	60	24.63	110.55	4.49	135.46
Hg	0.111	*0.163*	1.47	*0.19*	0.2	0.15	0.5	0.036	0.122	3.39	0.15
C**	101.69	87.45	0.9	95.22	NA		NA	53.84	70.8	1.3	43.63

*Includes data points pre 1850; **includes values from 1940–2014 and was multiplied by 1000

Except V, Mn, and Fe, the trace elemental concentrations show strong positive Pearson correlations among each other (Fig. S2).

In Lake Llaviucu, the average fluxes in post-1950 sediments relative to the average fluxes in pre-1950 sediments (post-1950 vs. pre-1950, Table 2) range mostly from a factor of 2.5 (Fe) to 4.5 (Pb), i.e., slightly higher than those observed in Lake Fondococha (2.1 to 4.4; Table 1, Fig. 2b). However, the total fluxes (post-1950) are much higher in Lake Llaviucu compared to the fluxes calculated for Lake Fondococha (factor of 3.2–29.5, Fe < Cd; Table 3). Trace element fluxes determined in the topmost part of Lake Llaviucu’s and Lake Fondococha’s sediments are similar to trace element fluxes determined from the annually integrating sediment traps (2014–2015, Tab. S10), except for Fe and Mn, where the sediment traps recorded much higher values.

**Table 3:** Comparison of total concentrations and fluxes between the two lakes (LLA—Lake Llaviucu; FON—Lake Fondococha) for pre-1950 and post-1950 values. Values are sorted after post-1950 fluxes

Trace element	Concentrations: LLA/FON		Fluxes: LLA/FON	
	Pre-1950	Post-1950	Pre-1950	Post-1950
Fe	0.77	0.79	3.55	3.24
Mn	0.73	0.88	3.38	3.59
Hg	1.18	1.06	5.54	4.27
Ni	0.92	1.28	4.25	5.19
V	1.10	1.30	5.08	5.26
Cu	1.29	1.64	5.95	6.70
Co	1.07	1.67	4.96	6.84
Cr	1.62	1.97	7.53	7.98
As	1.83	2.26	8.44	9.37
Zn	2.34	3.06	10.83	12.54
Pb	3.38	6.26	15.63	26.11
Cd	4.99	7.13	23.09	29.49

### Apportionment of trace elements into different sources

#### Changes in the sources of trace elements and Hg measured in the sediments of Lake Fondococha since 1760 ad

**Fig. 4:**
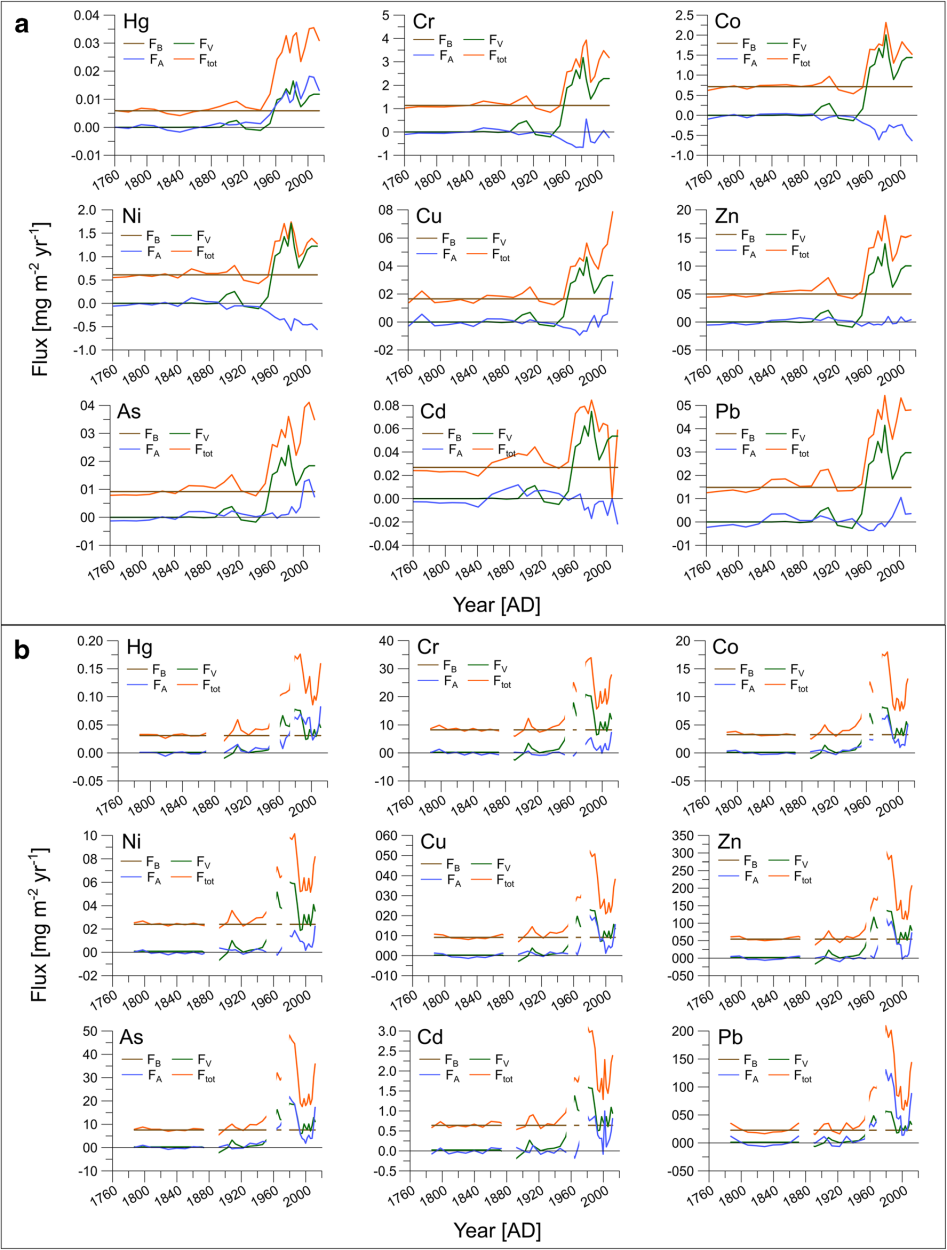
Apportionment of total trace element fluxes. Background (pre-1900) fluxes (*F*_B_), erosion-related fluxes (*F*_V_), airborne fluxes (*F*_A_), and total fluxes (*F*_tot_) for different trace elements measured in **a** Lake Fondococha ca. 1760–2014 and **b** Lake Llaviucu ca. 1785–2014. Please note that white gaps refer to the outliers presented in Fig. 3b. These samples were excluded from the calculations. Apportionments of further trace elements are presented in Fig. S3

The apportionment of the Hg and other trace element fluxes (*F*_tot_, *F*_B_, *F*_V_, and *F*_A_) measured in remote Lake Fondococha between ca. 1760 and 2014 is shown in Fig. 4a and Tab. S6. Remarkably, and for all investigated elements, the pre-1850, the pre-1900, and the pre-1950 total fluxes (*F*_tot_) are constant, indicating that *F*_B_ (defined here as pre-1900 *F*_tot_ values) is a robust estimate of ambient background conditions. Between ca. 1760 and 1950, contributions attributable to variable soil erosion and lithogenic flux (*F*_V_) are low for Cr, Co, Ni, Cu, Zn, As, and Pb (~ 3.5%, Fig. 4a, Tab. S6) except for a small positive peak at around 1910. Also, their atmospheric and point source contributions (*F*_A_) remain insignificant until 1950. For Cd and Hg, both *F*_V_ and *F*_A_ (3.1%, resp. 5.2–5.9%; Fig. 4a, Tab. S6) show very low values, but in contrast to the other trace elements, *F*_A_ is higher than *F*_V_.

From 1950 to 2014, rapid increases of *F*_tot_ are observed for Cr, Co, Ni, Cu, Zn, As, Cd, Hg, and Pb. More than 85% of the post-1950 *F*_tot_ of the trace elements is attributable to enhanced soil erosion (*F*_V_) and background fluxes (*F*_B_, Tab. S6). In the case of Hg, *F*_V_ and *F*_B_ contribute ~ 58% to the post-1950 *F*_tot_ (Tab. S6). Between ca. 1970 and 1990, lithogenic contributions (*F*_V_) exceed background fluxes (*F*_B_) by a factor of approximately 2 and are the dominant source of all studied elements in Lake Fondococha’s sediments.

Atmospheric contributions (*F*_A_) in the past two decades (from 1996 to 2014) show negative values for Cr, Co, Ni, Zn, and Cd. Copper (15.7%), As (26.2%), and Pb (12.2%) show significantly higher *F*_A_ contributions. *F*_A_ of Cu shows additionally a rapid increase over the last decade, in contrast to the other trace elements measured in Lake Fondococha. For Hg, *F*_A_ contributes with 42.2% at slightly higher importance as *F*_V_ (37.03%) to the post-1950 deposition. In the period from 1970 to 1990, *F*_A_ (39.1%) is equally important as *F*_V_ (41.1%). In the last two decades (from 1996 to 2014), the *F*_A_ of Hg (48.2%) becomes higher than the *F*_V_ (33.6%, Tab. S6). Moreover, the increasing trend of *F*_A_ starts much earlier than for Cu, As, and Pb.

#### Relative contributions of different sources to the trace element and Hg content in Lake Llaviucu’s sediments since 1780 ad

The relative importance of the pre-1950 trace element sources measured in peri-urban lake Llaviucu (Fig. 4b) compares well with those determined in Lake Fondococha. Comparably low pre-1850, pre-1900, and pre-1950 fluxes in Lake Llaviucu suggest that *F*_B_ (from here on average pre-1900 fluxes) is representative and reflects background conditions. Therefore, *F*_B_ contributes with values up to 100% the most to the *F*_tot_ of Cr, Co, Ni, Cu, Zn, As, Cd, and Pb between ca. 1760 and 1950 (Tab. S7). Anthropogenic point sources and atmospheric input (*F*_A_) contribute only between 2 and 4.5% to *F*_tot_ of Co, Cu, As, and Pb. The lithogenic fractions (*F*_V_) are negligible (Tab. S7). *F*_A_ of Hg is slightly higher and contributes approximately 8.9% to *F*_tot_ (Tab. S7). Pre-1950 *F*_A_ (point sources and atmospheric contribution) of Hg calculated for Lake Llaviucu is slightly higher than in Lake Fondococha (5.9%; Tab. S6). Interestingly, the temporary *F*_tot_-peak (driven by an increase in *F*_V_) at around 1910 occurs also in the profiles of Lake Llaviucu and is synchronic to that observed in Lake Fondococha.

The general picture of the post-1950 trace element sources in Lake Llaviucu, on the other hand, differs from remote Lake Fondococha (Fig. 4b): the non-lithogenic fraction (*F*_A_) of most of the trace elements is substantially larger than in Lake Fondococha and shows positive values for all trace elements. *F*_A_ accounts for 7.5–8.8% (V < Cr < Ni), or 16.3–28.8% (Cd < Zn < Co < Cu), or 35.8–53.8% (As < Hg < Pb) of *F*_tot_ (total fluxes; Tab. S7). Lithogenic sources (*F*_V_) contribute from 24.7 to 49.5% to the total fluxes (*F*_tot_, Tab. S7). The high *F*_A_ levels of As, Hg, and Pb peaked in the late 1960s and between 1980 and 1990 (*F*_A_ > 47.7% of *F*_tot_; Tab. S7). The drastic increase in *F*_A_ of Cu as observed in Lake Fondococha is not detected in Lake Llaviucu. However, in contrast to the pattern observed in Lake Fondococha, *F*_tot_ values measured in Lake Llaviucu do show an increasing trend after the year 2000. This trend is mostly coinciding with an increase in *F*_A_ for all the trace elements presented in Fig. 4.

## Discussion

We investigated the differences in Hg and other trace element depositions in two Ecuadorian Andean lakes, remote Lake Fondococha (4130 m a.s.l.) and peri-urban Lake Llaviucu (3140 m a.s.l.). It appears from Figs. 2 and 3 that variations in mass accumulation rates (MAR) are an important driver for the patterns and changes observed in the total fluxes. The element fluxes measured in Lake Llaviucu’s and Lake Fondococha’s sediment traps (Tab. S10), however, are comparable with those calculated for the most recent sediments. This suggests that the fluxes estimated for the down-core sediments are plausible.

The correlation coefficients among the most solid trace elements (Lake Fondococha: Fig. S1; Lake Llaviucu: Fig. S2) indicate a common source and common transport process of trace elements to the sediments. Only Hg and As show significant (*p* < 0.05) positive correlations (Hg: *r* > 0.84; As: *r* > 0.56) with the concentrations of elements typical for organic matter (C, N, and S, Fig. S1), suggesting that their sources, cycling, and fate are partly linked to those of sedimentary organic matter.

Given South America’s long history of trace element pollution (in particular Hg; Cooke and Abbott 2008; Cooke and Bindler 2015; Engstrom et al. 2014), it is likely that pre-1900 (similar to the pre-1800 and pre-1850) background levels (*F*_B_) also contain an anthropogenic contribution. Our profiles, however, are too short to detect the undisturbed, natural background values. This in fact might affect the presented apportionment of fluxes (*F*_B_, *F*_V_, and *F*_A_).

### Trace elements in Lakes Fondococha and Llaviucu

#### Pre-1950 trace element fluxes in the sediments of Lakes Fondococha and Llaviucu

The pre-1950 trace element fluxes in both lakes were very constant except for the synchronic temporary peaks observed at around 1910. An obvious interpretation of such synchronous peaks in trace element fluxes might be the large scale deposition of volcanic tephra layers of nearby volcanoes (Konieczka et al. 2018; Arcusa et al. 2020). The results of tephrostratigraphic analyses reported by Arcusa et al. (2020), however, did not reveal any evidence of macro, nor crypto tephra layers in the two sediment sequences during this time. The apportionment of the total flux (*F*_tot_) peaks, on the other hand, revealed that in both lakes *F*_V_ (lithogenic, erosion) dominated. Bandowe et al. (2018) reported elevated concentrations and fluxes of low molecular weight (LMW) polycyclic aromatic compounds (PACs) in this lake during the same period (shown for Lake Fondococha in Fig. S4). LMW-PACs are indicative of biomass burning (Bandowe et al. 2018). Therefore, we suggest that these fires induced disturbance of the Páramo vegetation (Lake Fondococha), and shrubs and grassland (Lake Llaviucu) which, in turn, increased the erosion of the soils rich in volcanic material in their watersheds (*F*_V_). Consequently, in the period of 1760 to 1950, the two lake systems were mainly affected by variations caused directly in their watersheds (changes in *F*_V_).

#### Post-1950 trace element and Hg fluxes in Lake Fondococha’s sediments

The apportionment of trace element and Hg fluxes (*F*_B_, *F*_V_, *F*_A_, and *F*_tot_) in remote high-altitude Lake Fondococha revealed that the post-1950 fluxes (until ca. 1995) are dominated by variations in *F*_V_ (except for Hg, where *F*_A_ > *F*_V_). The *F*_V_ patterns for the trace elements (except Hg) in Lake Fondococha follow again similar trends as observed in the LMW PAC-reconstruction from Bandowe et al. (2018; Fig. S4). This might indicate that local anthropogenic activity (e.g., slash and burn techniques that lead to increased erosion) was still at a high level and may have dominated over other sources (*F*_A_) of trace elements. The short-term relative drop by 20% in *F*_tot_ (mostly caused by a drop in *F*_V_) can be attributed to restrictions in land use coming along with the establishment of the recreational area in 1977. In the cases of Cu, As, and Pb, the relative short-term *F*_V_ decrease during the 1980–1990s is compensated by a strong relative increase of *F*_A_. We consider the opening of the Cuenca-Molleturo-Naranjal highway in 1991 with a traffic of currently more than 900,000 vehicles per year (data of 2012 from ETAPA EP) as a possible source for excess Cu and Pb (Hewitt and Candy 1990; Das et al. 2015). This increasing trend measured in Lake Fondococha’s sediments occurred synchronous to a rising in high molecular weight PACs (HMW; formed during high-temperature combustion of fossil fuels, e.g., vehicles and industries) and underlines the impact of this highway on the lake systems (Fig. S4; Bandowe et al. 2018). Additionally, *F*_A_ (point sources, and airborne) of Cu shows a rapid increase in the last decade, which could be further promoted by newly established mining areas around the park (Roy et al. 2018).

The situation is very different for Hg, where ~ 42.2% of the post-1950 deposition can be attributed to *F*_A_ (here presumably mostly atmospheric deposition; Tab. S6). Given the potentially long traveling time of Hg in the atmosphere (Schroeder and Munthe 1998; Phillips et al. 2011), sources from long-distance transport are plausible (Beal et al. 2014) and are possibly more important than local point sources. The sources of some PACs measured in Lake Fondococha (Bandowe et al. 2018 in Fig. S4) was similarly attributed to this mechanism (dominance of long-distance transported sources).

#### Post-1950 trace element and Hg fluxes in Lake Llaviucu’s sediments

The composition of the post-1950 trace element and Hg sources are more variable in peri-urban Lake Llaviucu compared to Lake Fondococha. Particularly, the high contribution of *F*_A_ (*F*_A_ regularly > 40% of *F*_tot_; Fig. 4b, Tab. S7) to *F*_tot_ of Co, Ni, Cu, Zn, Cd, and Pb between 1970 and 1990 is different from that observed in Lake Fondococha. We consider traffic (combustion, dust from tire-rubber and brakes-friction) and industrial dust from the metropolitan area of Cuenca as possible airborne sources (Hewitt and Candy 1990; Das et al. 2015). Intensive caged fishery (aquaculture, fish fodder (Cu), fertilizer, Xia et al. 2018; and antifouling (Cu) treatment of nets, Loucks et al. 2012) between 1978 and 1998 in Lake Llaviucu (data provided by ETAPA EP) might be an additional local point source of trace elements responsible for the observed excess fluxes (*F*_A_) during this period. For example, a systematic survey of different lakes in the central Yangtze River basin revealed a close positive relationship between aquaculture stocking size and measured trace element concentrations (Xia et al. 2018). Furthermore, we suggest that the different geography of Lake Llaviucu (e.g., microclimatology- located below cloud layer, large catchment-to-lake ratio- ~ 14 times higher than Lake Fondococha, and differences in vegetation cover) leads to higher (than Lake Fondococha) *F*_tot_ of the particle-bound elements (Cr, Co, Ni, Cu, Zn, Cd, and Pb) and also shapes the relative contributions of the different sources to the *F*_tot_. For example, the particle-bound trace elements can be scavenged in the cloud layer, wet-deposited, and lead to higher concentrations in Lake Llaviucu (and hence, would not reach Lake Fondococha). Mercury (mostly in gaseous phase; Phillips et al. 2011) on the other hand, shows a similar source composition as observed in Lake Fondococha (*F*_A_: 40.5%, Tab. S7) within the same period. This indicates that Hg is not efficiently scavenged by the cloud layer. However, the observed increase in *F*_A_ of Hg in Lake Llaviucu is also synchronic with the *F*_A_ of the other trace elements. This also could suggest that the amount of Hg deposited in Lake Llaviucu is more driven by the nearby urban activities than by long-distance transport.

**Fig. 5:**
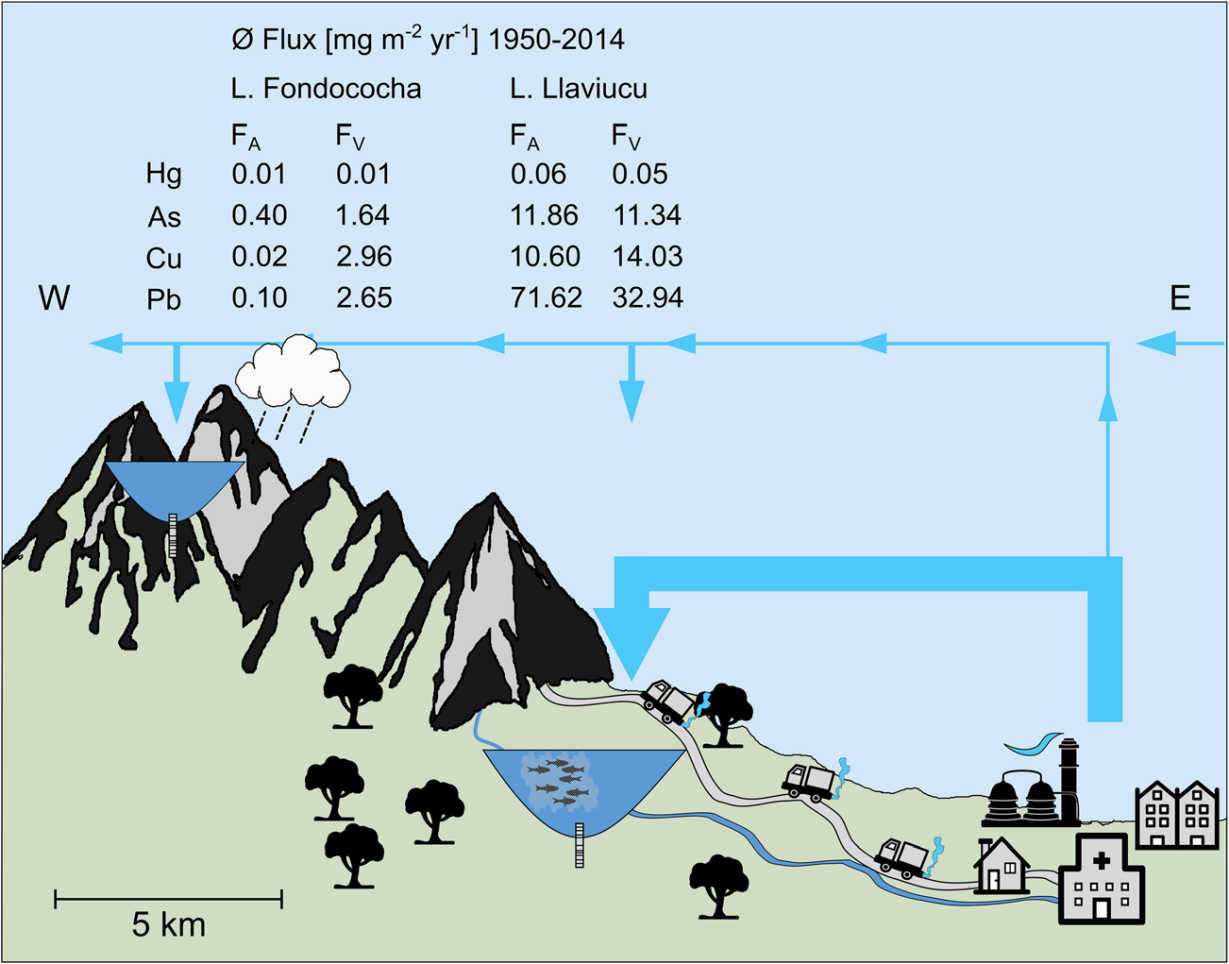
Sketch depicting the source to sink pathways of trace elements in Cajas National Park. Lake Llaviucu is the lower-elevation lake, located in the vicinity of the city of Cuenca and Lake Fondococha is situated at a relatively remote distance from Cuenca. The table represents average post-1950 fluxes of *F*_A_ (point sources and airborne fraction) and *F*_V_ (soil erosion). This figure was modified from Bandowe et al. (2018)

The comparison of the absolute post-1950 *F*_A_ fluxes of Hg, As, Cu, and Pb (all positive in both lakes) as presented in Fig. 5 reveals that differences in the geography (e.g., watershed size, cloud layer position, vegetation cover, vicinity to urban center) may affect the distribution of particle-bound trace elements more than the distribution of the mostly gaseous Hg. However, it is important to keep in mind that the local point sources (caged fishery) present in Lake Llaviucu’s watershed may bias this comparison.

#### Limitation of the implemented approach

We attribute negative *F*_A_ values (*F*_A_ = *F*_tot_ − *F*_B_ − *F*_V_), as observed for some of the trace elements in Lake Fondococha (post-1950; e.g., Cr, Co, Ni, Cd), to an artifact of the calculations after Perry et al. (2005). The total fluxes (*F*_tot_) are fixed and hence, do not directly influence *F*_A_. Therefore, negative *F*_A_ values result from the overestimation of either *F*_B_ or *F*_V_. Background fluxes (*F*_B_) can be overestimated due to (i) high MAR pre-1900 or (ii) high elemental concentrations pre-1900. However, the shapes of *F*_tot_ in Fig. 4 reveal that post-1950 *F*_tot_ values are generally higher than pre-1900 values (here the same as *F*_B_) and hence, *F*_B_ alone cannot explain negative values. Therefore, it is likely that *F*_V_ can be overestimated too. Perry et al. (2005) used a regional ^210^Pb-inventory encompassing ^210^Pb-profiles of different regional lakes and inferred a regional standard MAR to account for sedimentary focusing effects. Due to a lack of ^210^Pb-profiles in our study region, however, it was not possible to implement this in a similar way. Yet, this would not explain, why the approach worked for all elements in the case of Lake Llaviucu (no negative values) and for the other elements in Lake Fondococha. Therefore, we suggest that it must be a feature specific to Cr, Co, Ni, and Cd in Lake Fondococha’s watershed. One reason here could be that the watershed’s soil composition in the post-1950 period is depleted in Cr, Co, Ni, and Cd and that therefore, *F*_B_ of these trace elements would be overestimated. Furthermore, the cumulative uncertainties (RSD: ~ 11–16%; and variations in recovery rates post-1950 to pre-1950) of these specific elements could have resulted in the negative *F*_A_ values too. Keeping these caveats in mind, we therefore suggest that in the interpretation of the different source fractions presented in this study, emphasis should be placed on the tendencies and trends rather than on the absolute values.

### Regional, global comparison and implications

#### Trace element profiles

Peak fluxes of Co, Ni, Cu, Zn, As, and Cd measured in the sediments of Lake Fondococha are similar to those observed in Lake Chipian, a high-altitude (4350 m a.s.l) lake in the vicinity (~ 15 km) of Cerro de Pasco, a Peruvian city, which is famous for its mining activities (Cooke and Abbott 2008). However, trace element fluxes in Lake Chipian show the initial modern increases (around 1935–1940), and peak values (mid-1950s) earlier than Lake Fondococha (in the 1970s). In contrast to Lake Fondococha, Lake Chipian recorded decreasing fluxes of Cu, Zn, As, Cd, and Pb since the 1960s. The relative flux patterns observed in Lake Fondococha (except for Co, Ni, and As) is comparable with those reconstructed from Lake Pirhuacocha, a small high-altitude lake (4520 m a.s.l), which is nearby (11 km) a modern silver mine in the Peruvian Morococha mining region (Cooke and Abbott 2008). This lake recorded a similar strong onset in the 1950s and peak values at around 1975. A decrease towards 2000 (topmost sample in their study) is also comparable to Lake Fondococha’s trace element profile. Absolute flux values, however, were higher (2–33-fold) in the Peruvian lake. The temporal variations of Lake Llaviucu’s trace element profiles are mostly similar to those recorded in Lake Pirhuacocha (Cooke and Abbott 2008), but peak values are generally higher in Lake Llaviucu (up to a 5-fold).

Lake Fondococha’s trace elemental concentrations compare well with those from remote Lake Bolterskardet (Svalbard, Sun et al. 2006). Furthermore, post-1950 peak fluxes lay in the same order of magnitude (0.4–10-fold Pb < Cu) as those reconstructed from two remote high-altitude lakes in the center of the Adirondack mountains region (NY, USA, Sarkar et al. 2015). Here, differences can mostly be explained with differences in the bedrock composition, since *F*_V_ was the dominant source. Lake Llaviucu’s post-1950 peak values, however, are 13 (Pb) to 81 (Cu) times higher, representing the peri-urban influences.

Skordas et al. (2015) provide a compilation (we refer to their Table 2) of surface sedimentary heavy metal concentrations from different lakes in the northern hemisphere. Surface sediment concentrations of Cr, Ni, and Pb determined in Lake Fondococha are in the lower third, Cu in the medium range, and Co and Zn lay at the upper end (peak values in Lake Fondococha were slightly higher). Interestingly, maximum Zn and Pb concentrations in Lake Llaviucu’s sediments are a 1.2- to 1.5-fold higher than the highest reported in Skordas et al. (2015). The other elemental concentrations (except Ni) compare with the medium and high concentrations recorded in Skordas et al. (2015). We suggest that, besides differences in bedrock composition (volcanic material), mostly the point sources (caged fishery) and the proximity of Lake Llaviucu to urban activities caused such high concentrations.

#### Mercury profiles

The profiles of Hg fluxes from the two studied lakes (from ca. 1760 to 2014) show some differences to profiles reported from the northern hemisphere (mostly a delayed modern-era onset; Perry et al. 2005; Engstrom et al. 2014; Sarkar et al. 2015; Yang et al. 2016; Engels et al. 2018). The delayed onset of steep increases of Hg fluxes in recent times, however, was also observed in other sites from South America (Peru: Beal et al. 2013; Chile: Álvarez et al. 2018, Guédron et al. 2019; Hermanns and Biester 2013).

Noteworthy are the nearly constant pre-1950 Hg fluxes of 6.5 μg m^−2^ year^−1^ (Lake Fondococha) and 35.9 μg m^−2^ year^−1^ (Lake Llaviucu) to both lakes. This contrasts with most other records worldwide where a marked and steady increase in the Hg fluxes was recorded during this time. Even reconstructions from western Canadian Alpine lakes (Phillips et al. 2011) and Arctic lake systems (Outridge et al. 2007) recorded a continuous increase in Hg fluxes over this period.

Lake Fondococha’s record does not reflect the structure of historical global or regional Hg emissions (Streets et al. 2011). Apparently, Lake Fondococha is far enough away from areas with substantial local primary or secondary Hg anthropogenic emissions, such as cinnabar mining or historical Hg emissions related to amalgamation in silver or gold mines, located in southern Ecuador (Tarras-Wahlberg et al. 2000; Betancourt et al. 2005), Peru or Bolivia (Cooke and Abbott 2008; Cooke et al. 2009; Cooke and Bindler 2015). Similar observations were reported from three high-altitude lakes from Peru (PLS5, PLS8, and PLS12; Beal et al. 2013).

The record from Lake Fondococha reflects rather background levels of a well-mixed atmosphere in the tropics and long-distance transport in the pre-1950 period (~ 6.5 μg m^−2^ year^−1^ Hg; Table 3) and values of anthropogenic point and airborne sources (*F*_A_) Hg during the post-1950 increase (12 μg m^−2^ year^−1^) that are very similar to values found in other remote lake systems: high-elevation lakes in Rocky Mountains (Mast et al. 2010), Lake Challa (Tanzania), Lake El Junco (Galapagos), or Lake Tahoe (Sierra Nevada; Engstrom et al. 2014). The enhanced long-distance transported PAC-input measured in Lake Fondococha (compared to the one measured in Lake Llaviucu; Bandowe et al. 2018) further supports the finding that Hg in this lake has significant contributions from long-range transport sources.

The post-1950 period of Lake Fondococha resembles the profiles of Peruvian lakes (Beal et al. 2013), Lake Chungará (northern high-altitude Chilean Andes; Guédron et al. 2019), and rural Lake Pillo (south-central Chile, low-altitude; Álvarez et al. 2018). For example, Lake Fondococha’s peak Hg flux values observed in the post-1950 period (from 24 to 33 μg m^−2^ year^−1^) correspond very well to those seen in Lake Chungará during the industrial era. The relative pattern of rural Lake Pillo is similar too, but absolute values are up to ten times higher. Peri-urban Lake Llaviucu’s post-1950 Hg flux pattern on the other hand shows similarities to the one observed in urban Lake Señoraza (south-central Chile; Álvarez et al. 2018) with a temporary peak at around 1980 and a decrease thereafter. Absolute peak fluxes (from 160 up to 177 μg m^−2^ year^−1^) are comparable to those measured in Lake Señoraza, a lake which is highly influenced by urban activities.

The wind field in Cajas National Park with predominantly NE/E flow from the northern Amazon basin (Schneider et al. 2018) and the tropical Atlantic supports the view that transport of contaminated air from the hotspots of Hg emissions in Peru and Bolivia is very limited. This can also explain the differences observed in the Hg profile measured in Bolivian Altiplano (Guédron et al. 2018). It is also debatable to what extent historical atmospheric Hg loads from the northern hemisphere mid-latitudes (Perry et al. 2005) were mixed and transported to our study site in southern Ecuador. Interestingly, the uniquely low ^137^Cs fallout rates that were found in several lakes across Ecuador (Gunkel 2003; Bandowe et al. 2018) suggest that transport of air from the mid-latitudes of both hemispheres to the southern Andes of Ecuador is very limited. This would provide evidence for the existence of a chemical equator (a distinct atmospheric division between the northern hemisphere and the southern hemisphere) in this part of Ecuador (Hamilton et al. 2008).

Finally, the Hg record of Lake Fondococha, in particular *F*_A_ of Hg deposition, supports the view of Engstrom et al. (2014) that historical (here starting ca. 1760) atmospheric Hg loads were potentially smaller than previously thought (e.g., Streets et al. 2011). The increase of atmospheric (*F*_A_) Hg deposition between pre-1950 and the post-1950 periods by a factor of 4.4 (Hg_tot_; Hg_A_ = 31.6) in Lake Fondococha and 3.4 (Hg_tot_; Hg_A_ = 14.2) in Lake Llaviucu, respectively, is at the upper end of the range that has been observed elsewhere (Pirrone et al. 2010; Engstrom et al. 2014).

Our results confirm previous findings from Peru (Cooke and Abbott 2008) that showed that the long-distance atmospheric contribution of rather particulate trace elements in the high Ecuadorian Andes are negligible (except for weak contributions of volatile Hg). This stands in contrast to findings from other high-altitude lake systems as for example observed in the Eastern Tibetan Plateau (Bing et al. 2016).

## Conclusions

We present sedimentary trace element and Hg fluxes reconstructed from two Andean lakes in the Cajas National Park (Lake Fondococha: 4130 m a.s.l., and Lake Llaviucu: 3140 m a.s.l.). The two sedimentary reconstructions (approximately 250 years long) represent the first trace element records from this region.

The reconstructions from both lakes showed relatively stable pre-1950 trace element fluxes and concentrations. They compare well with each other, as well as with other profiles from South America. But they differ from other study sites reported on the northern hemisphere. The apportionment of the source contributions to the pre-1950 trace element fluxes revealed that pre-1950 fluxes mostly depended on changes directly caused in the watershed (e.g., loss of vegetation and increased erosion due to fires and land use). This agrees with the findings of the recently published polycyclic aromatic compound (PACs) reconstructions from Lake Fondococha (Bandowe et al. 2018).

Both lakes registered a strong increase in trace element fluxes between the late 1950s and 1960s. Differences in the post-1950 deposition patterns of lower-elevation and peri-urban Lake Llaviucu indicate that airborne fraction and local point sources started to become more important for Hg, Cu, As, and Pb. In remote high-altitude Lake Fondococha, post-1950 fluxes are mainly driven by changes in the catchment, except for As, Hg, Pb, and most recently also Cu.

Interestingly, the post-1950 fraction *F*_A_ (point-sources and atmospheric contribution) of Hg is similar in both lakes (Lake Fondococha: ~ 42.5%; Lake Llaviucu: ~ 40.5%). The Hg deposition pattern in remote Lake Fondococha reveals that long-distance atmospheric transport was an important source. A similar importance of long-range transport was also observed for the recent PAC reconstructions from Lake Fondococha (Bandowe et al. 2018). The *F*_A_ of the particle-bound trace elements, on the other hand, is much lower in the high-elevation lake compared to lower-elevation Lake Llaviucu (Lake Fondococha: < 13.5%; Lake Llaviucu: up to 54%). We explain differences in the *F*_A_ profiles with Lake Llaviucu’s vicinity to urban activities (traffic, industrial emissions) as well as its exposure to direct sources (caged fishery).

Most element fluxes measured in Lake Fondococha do compare with other remote lake systems (Andes, Arctic). Lake Llaviucu, on the other hand, recorded flux and concentrations comparable with values measured in lake sediments on the northern hemisphere. Even though the park regulations (1977) showed positive effects on both lakes (temporary drop in fluxes), the highway through the park and recently established mining areas around the park affected and can further impact these lake systems and potentially lift the concentrations above threshold levels (ecosystem health).

## Supplementary Information

ESM 1 (DOCX 1.95 mb)

## Data Availability

The data and calculations presented in this manuscript can be found online at https://boris.unibe.ch/ (BORIS, University of Bern, Switzerland)
