# Supplementary material for: 250-year records of mercury and trace element deposition in two lakes from Cajas National Park, SW Ecuadorian Andes
Source: Environ Sci Pollut Res Int. 2020 Dec 5;28(13):16227–43. doi: 10.1007/s11356-020-11437-0 (PMC7969545; doi:10.1007/s11356-020-11437-0)
Supplement: Supplementary file 1 — (DOCX 1.95 mb) [file 11356_2020_11437_MOESM1_ESM.docx]

**A 250-year record of mercury and trace elements deposition in lakes from Cajas National Park, SW Ecuadorian Andes**

**Supplementary data**

Tobias Schneider^1,2,3^*, Benjamin A. Musa Bandowe^1,2,4^, Moritz Bigalke^2^, Adrien Mestrot^2^, Henrietta Hampel^5,6^, Pablo V. Mosquera^7,8^, Lea Fränkl^1,2^, Giulia Wienhues^1,2^, Hendrik Vogel^1,9^, Wojciech Tylmann^10^, Martin Grosjean^1,2^

1 Oeschger Centre for Climate Change Research, University of Bern, Hochschulstrasse 4, 3012 Bern, Switzerland

2 Institute of Geography, University of Bern, Hallerstrasse 12, 3012 Bern, Switzerland

3 Department of Geosciences, University of Massachusetts Amherst, 611 North Pleasant Street, Amherst, MA 01003-9297, USA

4 Multiphase Chemistry Department, Max Planck Institute for Chemistry, Hahn-Meitner-Weg 1, 55128 Mainz, Germany

5 Facultad de Ciencias Químicas, Universidad de Cuenca, Cuenca, Ecuador

6 Laboratorio de Ecología Acuática, Departamento de Recursos Hídricos y Ciencias Ambientales, Universidad de Cuenca, Cuenca, Ecuador

7 Subgerencia de Gestión Ambiental, Empresa Pública Municipal de Telecomunicaciones, Agua potable, Alcantarillado y Saneamiento (ETAPA EP), Cuenca, Ecuador

8 Departament de Biologia Evolutiva, Ecologia i Ciències Ambientals, Universitat de Barcelona, Barcelona, Spain

9 Institute of Geological Sciences, University of Bern, Baltzerstrasse 1+3, 3012 Bern, Switzerland

10 Institute of Geography, Faculty of Oceanography and Geography, University of Gdansk, Bazynskiego 4, 80309 Gdansk, Poland

***Corresponding author**

Dr. Tobias Schneider

Department of Geosciences

627 North Pleasant Street

233 Morrill Science Center

University of Massachusetts

Amherst, MA 01003-9297

Email: [tobias.schneider@giub.unibe.ch](mailto:tobias.schneider@giub.unibe.ch), [tobiasschnei@umass.edu](mailto:tobiasschnei@umass.edu),

ORCID: http://orcid.org/0000-0002-1593-0273

**Co-authors email addresses & ORCIDs**

Benjamin A. Musa Bandowe: [Benjamin.Bandowe@mpic.de](mailto:Benjamin.Bandowe@mpic.de), https://orcid.org/0000-0003-0605-2285

Moritz Bigalke: [moritz.bigalke@giub.unibe.ch](mailto:moritz.bigalke@giub.unibe.ch), https://orcid.org/0000-0002-6793-6159

Adrien Mestrot: [adrien.mestrot@giub.unibe.ch](mailto:adrien.mestrot@giub.unibe.ch), https://orcid.org/0000-0002-4387-3886

Henrietta Hampel: [hennihampel@gmail.com](mailto:hennihampel@gmail.com), https://orcid.org/0000-0003-2249-5369

Pablo V. Mosquera: [bmosquer@etapa.net.ec](mailto:bmosquer@etapa.net.ec), http://orcid.org/0000-0002-3974-6489

Lea Fränkl: [lea.fraenkl@gmail.com](mailto:lea.fraenkl@gmail.com), --

Giulia Wienhues: [giulia.wienhues@giub.unibe.ch](mailto:giulia.wienhues@giub.unibe.ch), https://orcid.org/0000-0002-4282-488X

Hendrik Vogel: [hendrik.vogel@geo.unibe.ch](mailto:hendrik.vogel@geo.unibe.ch), https://orcid.org/0000-0002-9902-8120

Wojciech Tylmann: [wojciech.tylmann@ug.edu.pl](mailto:wojciech.tylmann@ug.edu.pl), https://orcid.org/0000-0003-1749-5882

Martin Grosjean: [martin.grosjean@oeschger.unibe.ch](mailto:martin.grosjean@oeschger.unibe.ch), <http://orcid.org/0000-0002-3553-8842>

Number of pages: 13

Number of figures: 4

Number of tables: 10

**
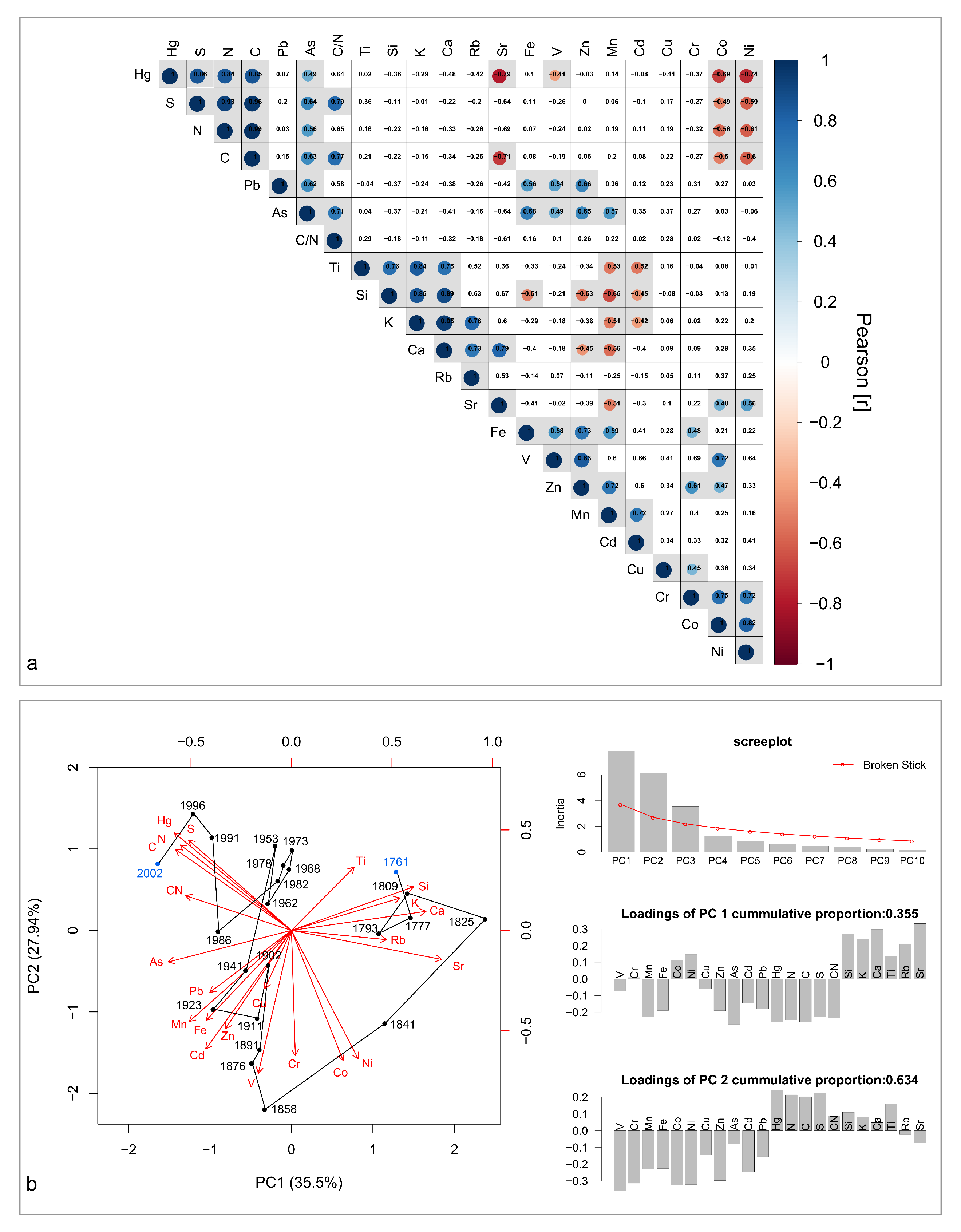
**

Fig. S1 Pearson correlations matrix and PCA for trace elements of Lake Fondococha. [a] Pearson correlation matrix (α= 0.05) of all elements (concentrations), C, N, S and C/N, and XRF data (Si, K, Ca, Ti, Rb, Sr) of Lake Fondococha. Blank squares are not significantly correlated. [b] Principal component analysis conducted on centered values (the same elements as for the correlation matrix) of Lake Fondococha. Left panel shows the Biplot of PC1 and PC2, blue numbers highlight the start/end of the time series, the black line connects all samples chronologically. The right panel shows the screeplot, and the loadings of PC1 and PC2. PC3 is also significant, but not shown in the biplot

**
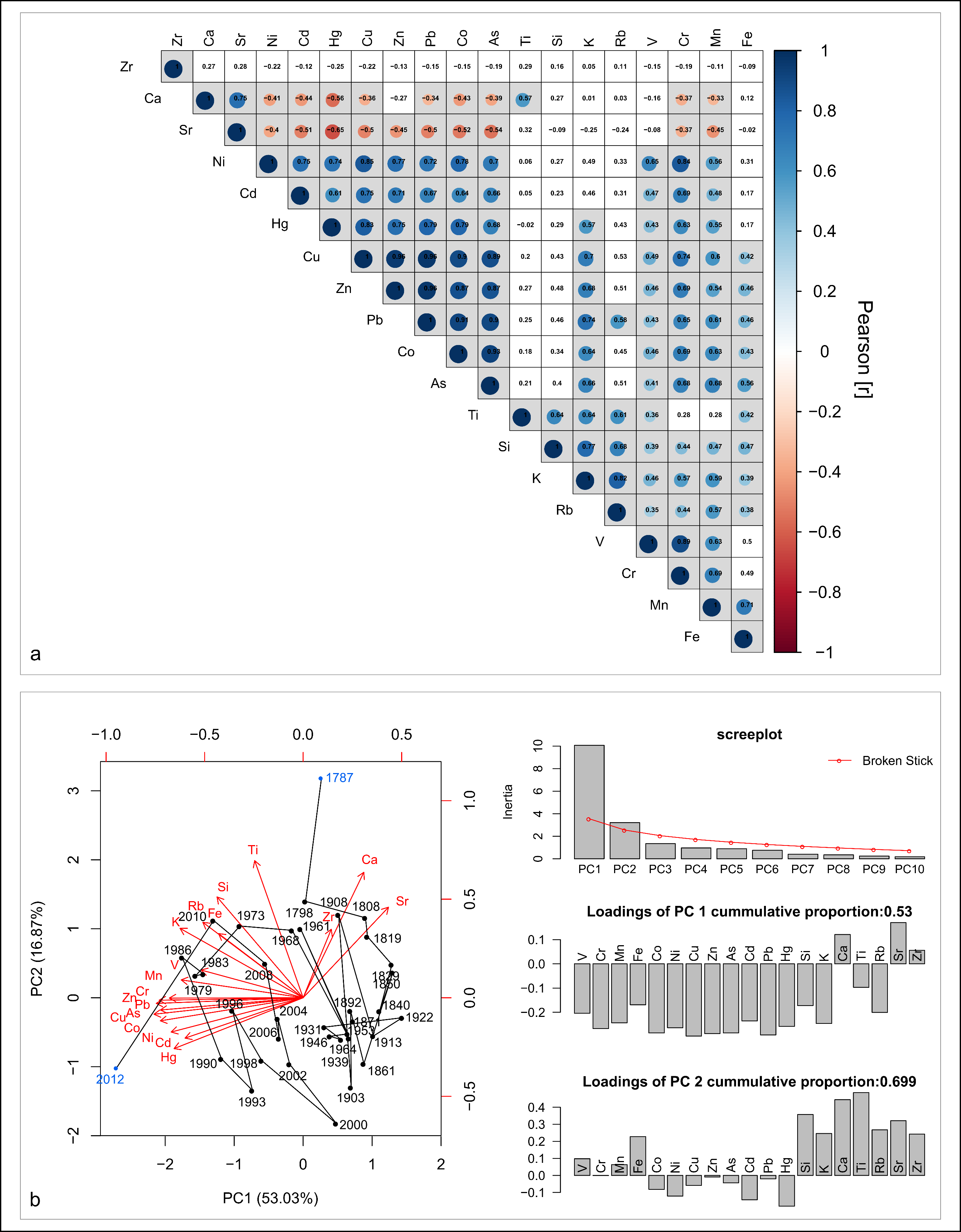
**

Fig. S2 Pearson correlations matrix and PCA for trace elements of Lake Llaviucu. [a] Pearson correlation matrix (α= 0.05) of all elements (concentrations) and XRF data (Si, K, Ca, Ti, Rb, Sr, Zr) of Lake Llaviucu. Blank squares are not significantly correlated. [b] Principal component analysis conducted on centered values (the same elements as for the correlation matrix) of Lake Llaviucu. Left panel shows the Biplot of PC1 and PC2, blue numbers highlight the start/end of the time series, the black line connects all samples chronologically. The right panel shows the screeplot, and the loadings of PC1 and PC2

**
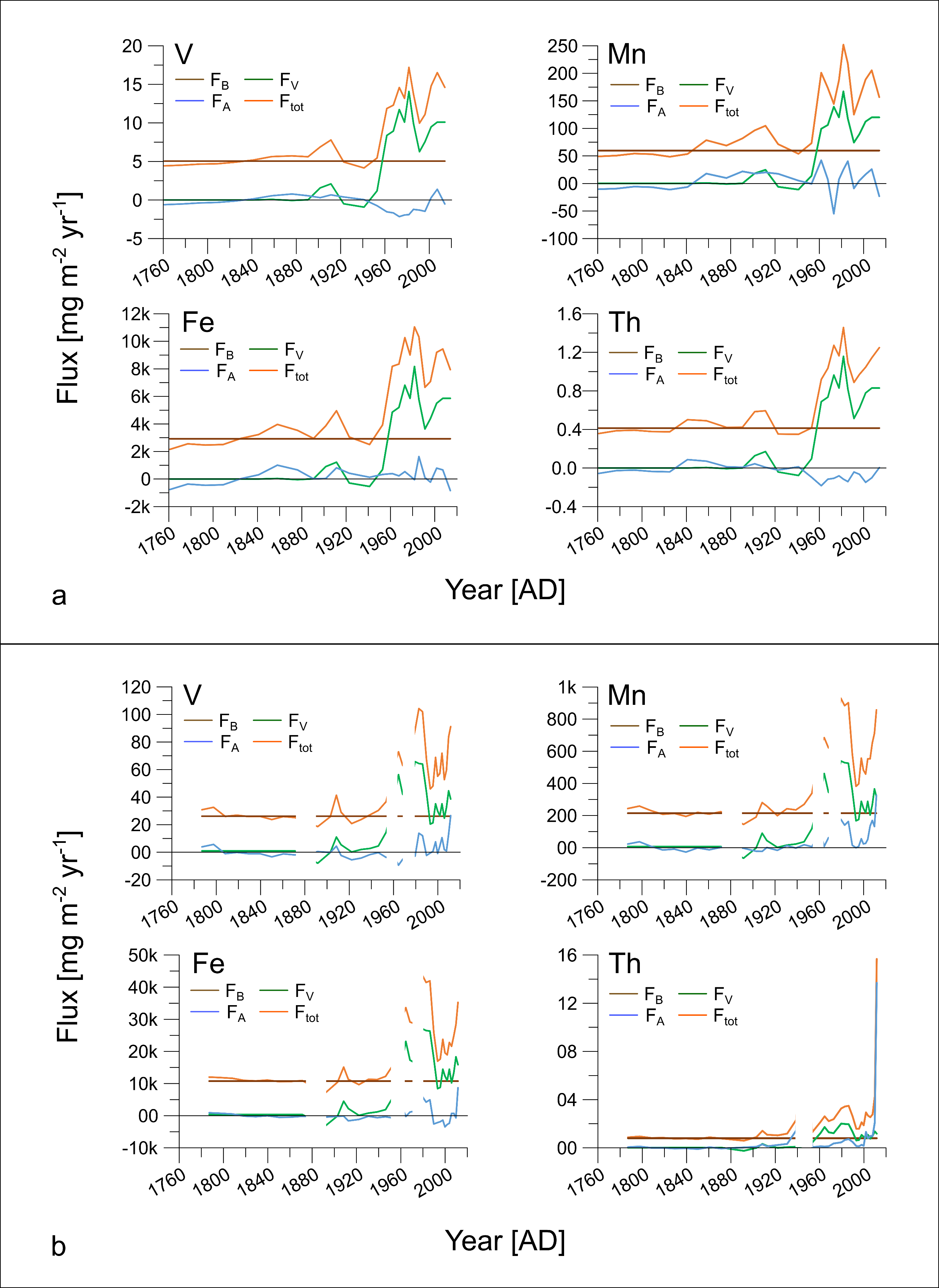
**

Fig. S3 Background (pre-1900) fluxes (F_B_), erosion-related fluxes (F_V_), airborne fluxes (F_A_), and total fluxes (F_tot_) of other elements (please refer to the plots) measured in [a] Lake Fondococha ca. 1760 – 2014 and [b] Lake Llaviucu ca. 1785 – 2014. Please note that white gaps refer to the outliers presented in Fig. 3 b. These samples were excluded from the calculations


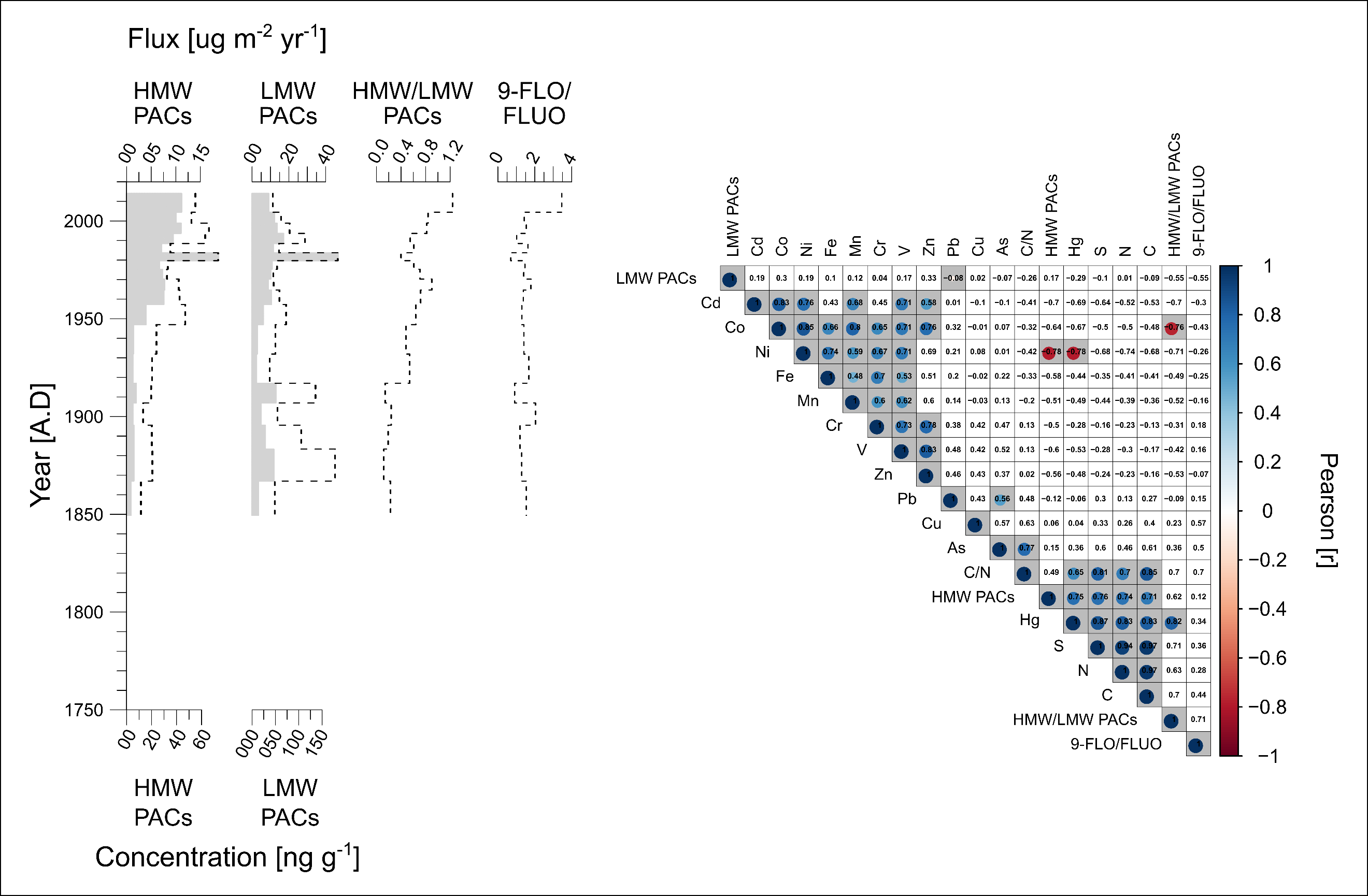


**Fig. S4** Polycyclic aromatic compounds (PACs) stratigraphy and ratios, and correlation matrix with trace elements and PACs for Lake Fondococha. Left panel: HMW: high molecular weight PACs, indicative of high temperature combustion (vehicles, industry); LMW: low molecular weight PACs, indicative of lower temperature combustion (biomass burning); HMW/LMW: ratio between the two sources, increase towards recent times indicates higher relative contribution of high temperature combustion derived compounds, resp. a relative decrease in the contribution of low temperature combustion derived PACs; 9-FLO/FLUO: ratio between 9-fluorenone/fluorene, higher values indicate higher contribution of long range transported PACs. Right panel: correlation matrix showing Pearson correlation matrix (α= 0.05) of standardized trace element and PACs data. Blank squares indicate not significantly correlated correlations. The dataset includes trace elements and PACs from ca. 1850 through 2014 and is shorter than the one in Fig. S1. The PACs-data is taken, incorporated, and replotted from Bandowe et al. (2018)

**Tab. S1** Microwave program used for the trace element sample digestion for both lakes.

| **Step** | **Time [min]** | **Power [W]** | **Temperature [°C]** |
| --- | --- | --- | --- |
| 1 | 5 | 300 | 120 |
| 2 | 10 | 700 | 200 |
| 3 | 30 | 450 | 200 |

**Tab. S2** ICP-MS operating parameters shown for both, trace element measurements and Hg measurement.

| **Parameter** | **Multi-element** | | **Mercury** | |
| --- | --- | --- | --- | --- |
| Gas mode | No Gas | He | No Gas | He |
| Rf Power [W] | 1550 | 1550 | 1550 | 1550 |
| Carrier Gas Flow Rate [l/min] | 1.05 | 1.03 | 1.05 | 1.05 |
| Nebulizer Pump [revolution/s] | 0.1 | 0.1 | 0.1 | 0.1 |
| Spray Chamber Temperature [°C] | 2 | 2 | 2 | 2 |
| Make Up Gas | 0 | 0 | 0 | 0 |
| *Lences* | | |  | |
| Extract 1 [V] | 0 | 0 | 0 | 0 |
| Extract 2 [V] | -180 | -200 | -180 | -160 |
| Omega Bias [V] | -95 | -100 | -95 | -95 |
| Omega Lens [V] | 9.3 | 10 | 9.3 | 8.4 |
| Cell Entrance [V] | -30 | -40 | -30 | -40 |
| Cell Exit [V] | -50 | -60 | -50 | -60 |
| Deflect [V] | 14.2 | 1 | 14.2 | 2.2 |
| Plate Bias [V] | -40 | -60 | -40 | -60 |
| *Cell* | | |  | |
| He Flow [ml/min] | 0 | 4.3 | 0 | 4.3 |
| H_2_ Flow [ml/min] | 0 | 0 | 0 | 0 |
| Octp Bias [V] | -8 | -8 | -8 | -18 |
| Octp Rf [V] | 200 | 180 | 200 | 190 |
| Energy Discrimination [V] | 5 | 5 | 5 | 3 |

**Tab. S3** Limit of detection (LOD) calculated from maximum blank values per trace element for both studied Lakes. LOD = detect. Limit [ug/l] * solution [l] * dilution coefficient (20) / sample weight [g] 🡪 ug/g = mg/kg. 0.1g was taken as the sample weight (in-weight per real sample).

| Trace  Element | Lake Fondococha | | | Lake Llaviucu | | |
| --- | --- | --- | --- | --- | --- | --- |
|  | Max. Blank [ug/l] | LOD  [ug/l] | LOD  [mg/kg] | Max. Blank  [ug/l] | LOD  [ug/l] | LOD  [mg/kg] |
| Al | 20.6861 | 20.6861 | 82.7445 | <0.128 | 0.1280 | 0.5120 |
| V | <0.023 | 0.0230 | 0.0920 | 0.0057 | 0.0057 | 0.0230 |
| Cr | <0.055 | 0.0550 | 0.2200 | 0.1320 | 0.1320 | 0.5279 |
| Mn | <0.092 | 0.0920 | 0.3680 | <0.003 | 0.0030 | 0.0120 |
| Fe | <0.472 | 0.4720 | 1.8880 | <0.397 | 0.3970 | 1.5880 |
| Co | <0.028 | 0.0280 | 0.1120 | 0.0014 | 0.0014 | 0.0056 |
| Ni | <0.102 | 0.1020 | 0.4080 | 0.0206 | 0.0206 | 0.0823 |
| Cu | <0.724 | 0.7240 | 2.8960 | 0.5282 | 0.5282 | 2.1128 |
| Zn | <0.159 | 0.1590 | 0.6360 | 1.1264 | 1.1264 | 4.5057 |
| As | <0.047 | 0.0470 | 0.1880 | <0.009 | 0.0090 | 0.0360 |
| Cd | <0.065 | 0.0650 | 0.2600 | <0.003 | 0.0030 | 0.0120 |
| Pb | <0.045 | 0.0450 | 0.1800 | <0.012 | 0.0120 | 0.0480 |
| Th | 0.0548 | 0.0548 | 0.2193 | 0.0240 | 0.0240 | 0.0959 |
| Hg | <0.004 | 0.0040 | 0.0080 | <0.004 | 0.0040 | 0.0080 |

**Tab. S4** Relative standard deviation (RSD) in percentage per trace element for Lake Fondococha based on triplicate (FON-06, and FON-20) and duplicate (FON-02, FON-03, and FON-14) measurements (one outlier FON-14c removed).

| Parameter | FON-02 | FON-03 | FON-06 | FON-14 | FON-20 | **MEAN** |
| --- | --- | --- | --- | --- | --- | --- |
| from [cm] | 0.00 | 0.50 | 2.00 | 6.00 | 9.10 | **-** |
| to [cm] | 0.50 | 1.00 | 2.50 | 6.50 | 9.60 | **-** |
| midpoint [cm] | 0.25 | 0.75 | 2.25 | 6.25 | 9.35 | **-** |
| in weight [mg] | 100.35 | 101.00 | 102.63 | 101.35 | 103.77 | **101.82** |
| Al [RSD%] | 13.00 | 20.83 | 9.04 | 1.03 | 3.84 | **9.55** |
| V [RSD%] | 12.35 | 19.72 | 8.69 | 2.88 | 3.32 | **9.39** |
| Cr [RSD%] | 15.93 | 23.31 | 28.94 | 3.70 | 6.45 | **15.67** |
| Mn [RSD%] | 12.77 | 19.93 | 16.69 | 5.43 | 3.04 | **11.57** |
| Fe [RSD%] | 12.98 | 20.87 | 15.61 | 4.28 | 2.96 | **11.34** |
| Co [RSD%] | 10.51 | 18.89 | 15.37 | 8.17 | 1.57 | **10.90** |
| Ni [RSD%] | 11.93 | 17.99 | 15.96 | 8.87 | 4.82 | **11.92** |
| Cu [RSD%] | 10.98 | 19.88 | 15.63 | 8.40 | 3.75 | **11.73** |
| Zn [RSD%] | 10.59 | 16.93 | 13.09 | 7.32 | 0.94 | **9.77** |
| As [RSD%] | 8.83 | 19.03 | 15.69 | 6.22 | 2.08 | **10.37** |
| Cd [RSD%] | NA | 12.26 | 17.54 | 24.96 | 7.62 | **15.59** |
| Pb [RSD%] | 0.73 | 6.08 | 6.41 | 17.81 | 5.36 | **7.28** |
| Th [RSD%] | 1.90 | 4.96 | 4.24 | 15.75 | 4.30 | **6.23** |

**Tab. S5** Recovery shown for the different trace elements per microwave batch (R1 – R7) and lake (LLA: Lake Llaviucu; FON: Lake Fondococha). The last column provides the certified concentrations [mg/kg] per element of the CRM that were used for the recovery calculation: 100/C_CRM_*C_measured_, where C: Concentration. The following CRM was used: 2709a San Joaquin Soil, National Institute of Standards and Technology (Mackey et al., 2010).

| Trace  Element | FON  R1 | FON  R2 | FON  R3 | FON  R4 | FON  R5 | FON  R6 | FON  R7 | **FON**  **Mean** | LLA  R1 | LLA  R2 | LLA  R3 | LLA  R4 | LLA  R5 | **LLA**  **Mean** | **CRM**  **[mg/kg]** |
| --- | --- | --- | --- | --- | --- | --- | --- | --- | --- | --- | --- | --- | --- | --- | --- |
| Al [%] | 61.96 | 61.86 | 78.37 | 71.93 | 74.04 | 65.76 | 69.05 | **69.00** | 52.20 | 46.93 | 43.07 | 51.33 | 50.45 | **48.79** | 73700 |
| V [%] | 91.90 | 91.28 | 119.62 | 110.34 | 108.61 | 102.36 | 107.11 | **104.46** | 78.68 | 71.25 | 67.16 | 79.44 | 74.62 | **74.23** | 110 |
| Cr [%] | 81.21 | 82.80 | 104.26 | 93.40 | 93.10 | 93.30 | 93.55 | **91.66** | 72.90 | 63.07 | 59.04 | 70.09 | 66.68 | **66.36** | 130 |
| Mn [%] | 97.72 | 96.75 | 134.23 | 122.91 | 120.75 | 123.80 | 130.58 | **118.11** | 87.58 | 85.85 | 84.06 | 85.05 | 83.71 | **85.25** | 529 |
| Fe [%] | 99.16 | 99.07 | 132.32 | 122.61 | 120.10 | 121.46 | 126.41 | **117.31** | 87.72 | 87.18 | 84.23 | 87.46 | 85.91 | **86.50** | 33600 |
| Co [%] | 97.72 | 96.99 | 130.65 | 119.53 | 114.67 | 117.87 | 122.96 | **114.34** | 83.59 | 81.27 | 79.04 | 85.22 | 80.88 | **82.00** | 12.8 |
| Ni [%] | 92.96 | 92.02 | 123.16 | 113.55 | 107.95 | 116.68 | 116.02 | **108.91** | 79.13 | 76.78 | 75.46 | 80.47 | 76.86 | **77.74** | 85 |
| Cu [%] | 88.24 | 87.62 | 118.18 | 109.83 | 103.02 | 107.58 | 111.75 | **103.75** | 80.24 | 76.94 | 75.05 | 79.44 | 75.42 | **77.42** | 33.9 |
| Zn [%] | 90.77 | 95.01 | 119.15 | 109.23 | 105.23 | 109.33 | 114.03 | **106.10** | 81.91 | 80.24 | 79.77 | 80.24 | 77.55 | **79.94** | 103 |
| As [%] | 91.49 | 95.19 | 123.97 | 115.07 | 111.86 | 111.24 | 116.47 | **109.33** | 81.08 | 78.49 | 74.58 | 82.81 | 77.06 | **78.80** | 10.5 |
| Cd [%] | NA | NA | 82.25 | NA | NA | 77.66 | NA | **NA** | 82.91 | 72.82 | 77.18 | 80.50 | 80.55 | **78.79** | 0.371 |
| Pb [%] | 70.72 | 67.39 | 87.34 | 79.41 | 68.19 | 79.38 | 72.05 | **74.93** | 60.42 | 57.07 | 55.06 | 60.36 | 57.87 | **58.15** | 17.3 |
| Th [%] | 81.02 | 77.42 | 98.46 | 93.56 | 77.51 | 87.23 | 80.44 | **85.09** | 81.88 | 78.10 | 76.06 | 103.68 | 87.57 | **85.46** | 10.9 |
| Hg [%] | 127.34 | 115.93 | 118.77 | 104.70 | NA | NA | NA | **116.69** | 65.35 | 62.06 | 58.73 | 60.42 | 60.48 | **61.41** | 0.9 |

NA: not available

**Tab. S6** Percentage of trace elements contributing sources to the total flux (F_tot_ = 100%) in discussed time periods presented for Lake Fondococha. F_B_= background proportion, F_V_= lithogenic fraction, F_A_= atmospheric and point sources.

| Element | Fraction | post-1950 [%] | pre-1950 [%] | 1970 – 1990 [%] | 1996 – 2014 [%] |
| --- | --- | --- | --- | --- | --- |
| V | F_B_ | 38.89 | 94.80 | 36.65 | 35.31 |
| V | F_V_ | 69.41 | 3.19 | 75.85 | 65.27 |
| V | F_A_ | -8.31 | 2.01 | -12.50 | -0.58 |
| Cr | F_B_ | 40.12 | 98.47 | 36.42 | 37.57 |
| Cr | F_V_ | 71.61 | 3.32 | 75.39 | 69.46 |
| Cr | F_A_ | -11.73 | -1.79 | -11.81 | -7.03 |
| Mn | F_B_ | 34.48 | 89.93 | 32.25 | 33.88 |
| Mn | F_V_ | 61.55 | 3.03 | 66.76 | 62.63 |
| Mn | F_A_ | 3.97 | 7.04 | 0.99 | 3.48 |
| Fe | F_B_ | 34.58 | 93.44 | 30.89 | 34.72 |
| Fe | F_V_ | 61.71 | 3.15 | 63.94 | 64.18 |
| Fe | F_A_ | 3.71 | 3.42 | 5.17 | 1.10 |
| Co | F_B_ | 43.97 | 98.93 | 39.73 | 43.66 |
| Co | F_V_ | 78.48 | 3.33 | 82.23 | 80.70 |
| Co | F_A_ | -22.46 | -2.26 | -21.95 | -24.36 |
| Ni | F_B_ | 47.02 | 100.47 | 41.81 | 48.47 |
| Ni | F_V_ | 83.92 | 3.38 | 86.55 | 89.60 |
| Ni | F_A_ | -30.94 | -3.85 | -28.36 | -38.08 |
| Cu | F_B_ | 35.75 | 97.09 | 35.11 | 29.61 |
| Cu | F_V_ | 63.81 | 3.27 | 72.67 | 54.74 |
| Cu | F_A_ | 0.43 | -0.36 | -7.78 | 15.65 |
| Zn | F_B_ | 36.02 | 94.53 | 32.70 | 34.40 |
| Zn | F_V_ | 64.30 | 3.18 | 67.68 | 63.58 |
| Zn | F_A_ | -0.32 | 2.29 | -0.38 | 2.02 |
| As | F_B_ | 31.11 | 93.58 | 30.78 | 25.91 |
| As | F_V_ | 55.52 | 3.15 | 63.71 | 47.89 |
| As | F_A_ | 13.37 | 3.27 | 5.51 | 26.20 |
| Cd | F_B_ | 39.92 | 91.67 | 36.30 | 43.14 |
| Cd | F_V_ | 71.25 | 3.09 | 75.14 | 79.74 |
| Cd | F_A_ | -10.37 | 5.24 | -11.44 | -20.61 |
| Pb | F_B_ | 35.03 | 94.12 | 32.83 | 30.81 |
| Pb | F_V_ | 62.51 | 3.17 | 67.95 | 56.96 |
| Pb | F_A_ | 2.46 | 2.71 | -0.77 | 12.23 |
| Th | F_B_ | 39.27 | 95.97 | 35.25 | 37.56 |
| Th | F_V_ | 70.09 | 3.23 | 72.96 | 69.44 |
| Th | F_A_ | -9.36 | 0.80 | -8.21 | -7.00 |
| Hg | F_B_ | 20.75 | 91.06 | 19.84 | 18.19 |
| Hg | F_V_ | 37.03 | 3.07 | 41.06 | 33.63 |
| Hg | F_A_ | 42.23 | 5.87 | 39.10 | 48.18 |

**Tab. S7** Percentage of trace elements contributing sources to the total flux (F_tot_ = 100%) in discussed time periods presented for Lake Llaviucu. F_B_= background proportion, F_V_= lithogenic fraction, F_A_= atmospheric and point sources.

| Element | Fraction | post-1950 [%] | pre-1950 [%] | 1970 – 1990 [%] | 1996 – 2014 [%] |
| --- | --- | --- | --- | --- | --- |
| V | F_B_ | 43.00 | 108.01 | 36.14 | 53.48 |
| V | F_V_ | 49.46 | -6.18 | 53.30 | 35.71 |
| V | F_A_ | 7.54 | -1.83 | 10.55 | 10.81 |
| Cr | F_B_ | 42.49 | 107.54 | 34.54 | 54.09 |
| Cr | F_V_ | 48.87 | -6.15 | 50.94 | 36.12 |
| Cr | F_A_ | 8.64 | -1.39 | 14.53 | 9.79 |
| Mn | F_B_ | 40.10 | 107.55 | 32.46 | 50.36 |
| Mn | F_V_ | 46.12 | -6.15 | 47.87 | 33.62 |
| Mn | F_A_ | 13.78 | -1.40 | 19.68 | 16.02 |
| Fe | F_B_ | 43.06 | 107.22 | 33.55 | 59.43 |
| Fe | F_V_ | 49.52 | -6.13 | 49.48 | 39.68 |
| Fe | F_A_ | 7.42 | -1.08 | 16.97 | 0.90 |
| Co | F_B_ | 33.87 | 103.60 | 25.47 | 47.22 |
| Co | F_V_ | 38.96 | -5.93 | 37.56 | 31.53 |
| Co | F_A_ | 27.17 | 2.33 | 36.96 | 21.24 |
| Ni | F_B_ | 42.44 | 106.01 | 33.50 | 54.52 |
| Ni | F_V_ | 48.81 | -6.06 | 49.41 | 36.40 |
| Ni | F_A_ | 8.75 | 0.06 | 17.09 | 9.07 |
| Cu | F_B_ | 33.14 | 101.88 | 22.55 | 45.29 |
| Cu | F_V_ | 38.11 | -5.83 | 33.26 | 30.24 |
| Cu | F_A_ | 28.75 | 3.95 | 44.19 | 24.48 |
| Zn | F_B_ | 34.61 | 106.20 | 23.13 | 49.61 |
| Zn | F_V_ | 39.80 | -6.07 | 34.11 | 33.12 |
| Zn | F_A_ | 25.59 | -0.12 | 42.76 | 17.27 |
| As | F_B_ | 29.86 | 102.05 | 21.03 | 43.68 |
| As | F_V_ | 34.35 | -5.84 | 31.01 | 29.16 |
| As | F_A_ | 35.79 | 3.79 | 47.97 | 27.16 |
| Cd | F_B_ | 38.91 | 107.74 | 26.87 | 49.38 |
| Cd | F_V_ | 44.75 | -6.16 | 39.63 | 32.97 |
| Cd | F_A_ | 16.34 | -1.57 | 33.49 | 17.65 |
| Pb | F_B_ | 21.50 | 101.16 | 12.82 | 32.23 |
| Pb | F_V_ | 24.73 | -5.79 | 18.90 | 21.52 |
| Pb | F_A_ | 53.77 | 4.63 | 68.28 | 46.26 |
| Th | F_B_ | 31.41 | 59.17 | 31.26 | 27.21 |
| Th | F_V_ | 36.12 | -3.38 | 46.10 | 18.17 |
| Th | F_A_ | 32.47 | 44.21 | 22.64 | 54.62 |
| Hg | F_B_ | 27.65 | 96.61 | 21.28 | 33.90 |
| Hg | F_V_ | 31.81 | -5.53 | 31.38 | 22.63 |
| Hg | F_A_ | 40.54 | 8.91 | 47.34 | 43.47 |

**Tab. S8** Lake Fondococha: TE (trace element), concentrations [µg g^-1^_ds_] and fluxes [mg m^-2^ yr^-1^] (pre-1950 and post-1950, recovery corrected values, for not recovery corrected values refer to Tab. 1), Factor (average in post-1950 sediments relative to the average in pre-1950 sediments), Recent (2 top samples, mean year: AD 2007), comparison with threshold effect concentrations (LEL: Low Effect Level, Persaud et al. 1993; ERL: Effect Range-Low, Long and Morgan 1991) from sediment quality guidelines (SQG) in MacDonald et al. (2000), and comparison with threshold values in agricultural soils (TAS) after Toth et al. (2016) for trace elements in Lake Fondococha (**bold**: concentrations exceed LEL values of SQG; *italics*: concentrations exceed ERL values of SQG)

| TE | This study  Concentration [µg g^-1^_ds_] | | | | SQGs-LEL  [µg g^-1^_ds_] | | TAS  [µg g^-1^_ds_] | This study  Flux [mg m^-2^ yr^-1^] | | | |
| --- | --- | --- | --- | --- | --- | --- | --- | --- | --- | --- | --- |
|  | Pre-1950^*^ | 1950 –2014 | Factor | Recent | LEL | ERL |  | Pre-1950^*^ | 1950 –2014 | Factor | Recent |
| Cr | 17.61 | 18.03 | 1.02 | 19.6 | 26 | 80 | 100 | 1.21 | 3.38 | 2.79 | 3.94 |
| Ni | 8.04 | 7.33 | 0.91 | 6.88 | 16 | 30 | 200 | 0.55 | 1.37 | 2.47 | 1.38 |
| Cu | **23.46** | **27.43** | 1.17 | **36.64** | 16 | 70 | 100 | 1.62 | 5.13 | 3.17 | 7.36 |
| Zn | 69.85 | 78.45 | 1.12 | 80.65 | 120 | 120 | 200 | 4.82 | 14.70 | 3.05 | 16.21 |
| As | **12.52** | **16.55** | 1.32 | **19.6** | 6 | 33 | 5.0 | 0.87 | 3.09 | 3.57 | 3.94 |
| Cd | 0.59 | NA | NA | NA | 0.6 | 5 | 1.0 | 0.04 | NA | NA | NA |
| Pb | 29.00 | **32.46** | 1.12 | **34.1** | 31 | 35 | 60 | 2.00 | 6.05 | 3.02 | 6.85 |
| Hg | 0.081 | 0.124 | 1.53 | 0.13 | 0.2 | 0.15 | 0.5 | 0.006 | 0.023 | 4.12 | 0.03 |
| C** | 79.95 | 102.44 | 1.3 | 117.89 |  | |  | 6.07 | 19.04 | 3.1 | 23.7 |

*Includes data points pre 1850; **multiplied by 1000; NA: not available

**Tab. S9** Lake Llaviucu: TE (trace element), concentrations [µg g^-1^_ds_] and fluxes [mg m^-2^ yr^-1^] (pre-1950 and post-1950, recovery corrected values, for not recovery corrected values refer to Tab. 2), Factor (average in post-1950 sediments relative to the average in pre-1950 sediments), Recent (2 top samples, mean year: AD 2011), comparison with threshold effect concentrations (LEL: Low Effect Level, Persaud et al. 1993; ERL: Effect Range-Low, Long and Morgan 1991) from sediment quality guidelines (SQG) in MacDonald et al. (2000), and comparison with threshold values in agricultural soils (TAS) after Toth et al. (2016) for trace elements in Lake Llaviucu (**bold**: concentrations exceed LEL values of SQG; *italics*: concentrations exceed ERL values of SQG)

| TE | This study  Concentration [µg g^-1^_ds_] | | | | SQGs-LEL  [µg g^-1^_ds_] | | TAS  [µg g^-1^_ds_] | This study  Flux [mg m^-2^ yr^-1^] | | | |
| --- | --- | --- | --- | --- | --- | --- | --- | --- | --- | --- | --- |
|  | Pre-1950^*^ | 1950 –2014 | Factor | Recent | LEL | ERL |  | Pre-1950^*^ | 1950 – 2014 | Factor | Recent |
| Cr | **41.04** | **45.06** | 1.10 | **47.2** | 26 | 80 | 100 | 13.16 | 34.30 | 2.61 | 36.93 |
| Ni | 10.39 | 11.52 | 1.11 | **12.66** | 16 | 30 | 200 | 3.32 | 8.72 | 2.63 | 9.9 |
| Cu | **41.22** | **52.21** | 1.27 | **57.77** | 16 | 70 | 100 | 13.22 | 39.97 | 3.02 | 45.15 |
| Zn | ***225.82*** | ***280.23*** | 1.24 | ***309.72*** | 120 | 120 | 200 | 72.29 | 215.53 | 2.98 | 242.17 |
| As | **32.94** | ***45.55*** | 1.38 | ***52.09*** | 6 | 33 | 5.0 | 10.55 | 35.21 | 3.34 | 40.65 |
| Cd | **2.65** | **3.37** | 1.27 | **3.55** | 0.6 | 5 | 1.0 | 0.85 | 2.56 | 3.03 | 2.78 |
| Pb | ***132.01*** | ***244.97*** | 1.86 | ***286.96*** | 31 | 35 | 60 | 42.35 | 191.16 | 4.51 | 224.21 |
| Hg | *0.185* | ***0.259*** | 1.40 | ***0.29*** | 0.2 | 0.15 | 0.5 | 0.060 | 0.194 | 3.24 | 0.23 |
| C** | 101.69 | 87.45 | 0.9 | 95.22 |  | |  | 53.84 | 70.08 | 1.3 | 43.63 |

*Includes data points pre 1850; **Includes values from 1940 – 2014 and was multiplied by 1000

**Tab. S10** Sediment trap data shown for Lakes Fondococha and Llaviucu. The sediment traps were deployed between summer 2014 and summer 2015. Details can be found in the main manuscript.

| Trace | Lake Fondococha | | Lake Llaviucu | |
| --- | --- | --- | --- | --- |
| Element | Conc. [mg kg^-1^] | Flux [mg m^-2^ yr^-1^] | Conc. [mg kg^-1^] | Flux [mg m^-2^ yr^-1^] |
| V | 79.28 | 26.59 | 110.10 | 47.37 |
| Cr | 22.58 | 7.57 | 33.75 | 14.52 |
| Mn | 6634.93 | 2224.94 | 3197.90 | 1375.88 |
| Fe | 91648.38 | 30733.15 | 72993.63 | 31405.25 |
| Co | 15.13 | 5.07 | 22.82 | 9.82 |
| Ni | 9.74 | 3.27 | 12.26 | 5.28 |
| Cu | 70.49 | 23.64 | 80.61 | 34.68 |
| Zn | 158.99 | 53.32 | 261.53 | 112.52 |
| As | 49.15 | 16.48 | 98.12 | 42.22 |
| Cd | 0.32 | 0.11 | 2.23 | 0.96 |
| Pb | 22.99 | 7.71 | 76.39 | 32.87 |
| Th | 4.22 | 1.42 | 1.91 | 0.82 |
